# Artemisinin Combination Therapies for Treatment of Uncomplicated Malaria in Uganda

**DOI:** 10.1371/journal.pctr.0010007

**Published:** 2006-05-19

**Authors:** Hasifa Bukirwa, Adoke Yeka, Moses R Kamya, Ambrose Talisuna, Kristin Banek, Nathan Bakyaita, John Bosco Rwakimari, Philip J Rosenthal, Fred Wabwire-Mangen, Grant Dorsey, Sarah G Staedke

**Affiliations:** 1 Uganda Malaria Surveillance Project, Kampala, Uganda; 2 Makerere University Medical School, Kampala, Uganda; 3 Ministry of Health, Kampala, Uganda; 4 Department of Medicine, San Francisco General Hospital, University of California, San Francisco, United States of America; 5 Institute of Public Health, Makerere University, Kampala, Uganda

## Abstract

**Objectives:**

To compare the efficacy and safety of artemisinin combination therapies for the treatment of uncomplicated falciparum malaria in Uganda.

**Design:**

Randomized single-blind controlled trial.

**Setting:**

Tororo, Uganda, an area of high-level malaria transmission.

**Participants:**

Children aged one to ten years with confirmed uncomplicated P. falciparum malaria.

**Interventions:**

Amodiaquine + artesunate or artemether–lumefantrine.

**Outcome Measures:**

Risks of recurrent symptomatic malaria and recurrent parasitemia at 28 days, unadjusted and adjusted by genotyping to distinguish recrudescences and new infections.

**Results:**

Of 408 participants enrolled, 403 with unadjusted efficacy outcomes were included in the per-protocol analysis. Both treatment regimens were highly efficacious; no recrudescences occurred in patients treated with amodiaquine + artesunate, and only two occurred in those treated with artemether–lumefantrine. However, recurrent malaria due to new infections was common. The unadjusted risk of recurrent symptomatic malaria was significantly lower for participants treated with artemether–lumefantrine than for those treated with amodiaquine + artesunate (27% versus 42%, risk difference 15%, 95% CI 5.9%–24.2%). Similar results were seen for the risk of recurrent parasitemia (51% artemether–lumefantrine versus 66% amodiaquine + artesunate, risk difference 16%, 95% CI 6.2%–25.2%). Amodiaquine + artesunate and artemether–lumefantrine were both well-tolerated. Serious adverse events were uncommon with both regimens.

**Conclusions:**

Amodiaquine + artesunate and artemether–lumefantrine were both highly efficacious for treatment of uncomplicated malaria. However, in this holoendemic area, despite the excellent performance of both regimens in terms of efficacy, many patients experienced recurrent parasitemia due to new infections. Artemether–lumefantrine was superior to amodiaquine + artesunate for prevention of new infections. To maximize the benefit of artemisinin combination therapy in Africa, treatment should be integrated with strategies to prevent malaria transmission. The impact of frequent repeated therapy on the efficacy, safety, and cost-effectiveness of new artemisinin regimens should be further investigated.

## Editorial Commentary

**Background:** Malaria parasites have become resistant in much of Africa to many commonly used treatments, such as chloroquine and sulfadoxine–pyrimethamine. Newer drugs, such as artemisinin-based combination therapies (ACTs), have been used extensively in Southeast Asia. Artemether–lumefantrine (an ACT) has now been adopted as first-line malaria treatment in Uganda, with the combination of amodiaquine and artesunate as a backup treatment. There are two ways that successful treatment is measured; first by whether the treatment works in curing the infection; and second by whether it prevents the disease recurring—either the same infection (known as recrudescence), or a new infection. The researchers wanted to look at how artemether–lumefantrine compared with amodiaquine and artesunate for treating symptomatic malaria, and also at whether there were any differences in recurrence, either of clinical malaria, or of parasite infection without symptoms, for 28 days after treatment.

**What this trial shows:** This randomized trial in young children with confirmed malaria in Uganda showed that both treatments resulted in a similar initial response to therapy, as measured by the risk of early treatment failure (within three days). The researchers found that artemether–lumefantrine was more effective at reducing the risk and delayed the time to recurrence of malaria, compared with amodiaquine and artesunate. Additionally, treatment with artemether–lumefantrine resulted in a reduced rate of parasite infection without malaria symptoms, as compared with amodiaquine and artesunate. Nearly all cases of recurrent malaria after either treatment combination arose from new infections, rather than from recrudescences.

**Strengths and limitations:** The trial was correctly designed to test the questions of interest, and enough patients were recruited to properly examine the relative effects of the two treatment combinations. However, patients were only followed up for 28 days after treatment. A longer follow-up period might have revealed a higher rate of recurrence of malaria after treatment. In the trial, artemether–lumefantrine was not administered with food, and it's known that lumefantrine is absorbed better when it is taken with a small amount of fat. It's possible that the effect of artemether–lumefantrine seen in the trial could have been an underestimate of what might be achieved in ideal conditions.

**Contribution to the evidence:** This trial adds information on the efficacy of artemether–lumefantrine as compared with amodiaquine and artesunate in East Africa for treatment of uncomplicated (i.e., non-severe) symptomatic malaria. The results are consistent with those of other trials on the efficacy of ACTs that have been performed in the region. The study also adds data on the risk of recurrent infections in an area where malaria occurs very frequently. Even following treatment with either ACT, the risk of recurrence was very high.

## INTRODUCTION

In Africa, widespread resistance of Plasmodium falciparum to chloroquine and sulfadoxine–pyrimethamine has required urgent introduction of alternative antimalarial therapies, including artemisinin-based combination therapies (ACTs) [1]. However, identifying appropriate regimens has been a challenging task [2]. In Southeast Asia, ACTs have been highly efficacious and associated with reductions in morbidity, gametocyte carriage, and malaria transmission [3,4]. However, there is relatively little experience with these drugs in Africa, where malaria transmission intensity is substantially greater and the pattern of antimalarial drug use is quite different [5,6].

In Uganda, chloroquine + sulfadoxine–pyrimethamine replaced chloroquine as the first-line recommended therapy for uncomplicated malaria in 2002 [7]. Drug efficacy studies conducted at eight sites around the country subsequently demonstrated quite poor efficacy of chloroquine + sulfadoxine–pyrimethamine [8–10]. As a result, artemether–lumefantrine was adopted as the new first-line antimalarial treatment in 2004, with amodiaquine + artesunate as a substitute, if artemether–lumefantrine was not readily available. However, this policy was adopted when little comparative efficacy and safety data on artemether–lumefantrine was available from Africa [11]. With shortages in drug supplies, limitations of available resources, and logistical issues, to date the new Ugandan drug policy has not been implemented. To inform antimalarial policy in Uganda, and to further investigate ACTs in Africa, we conducted a single-blind randomized clinical trial to compare the efficacy and safety of artemether–lumefantrine and amodiaquine + artesunate for the treatment of uncomplicated falciparum malaria in Tororo, an area of very high transmission intensity.

## METHODS

### Study Site

The study was conducted at Nagongera Health Centre, Tororo District, Uganda. At this rural site, malaria is holoendemic, occurring perennially with peaks following the two rainy seasons, from March to May and from August to September (Ugandan Ministry of Health, unpublished data, 1994). The entomological inoculation rate (number of infective bites per person per year), a measure of transmission intensity, was determined to be 591 in Nagongera, Tororo District (A. Talisuna, Uganda Ministry of Health, personal communication). The study protocol was approved by the Uganda National Council of Science and Technology and the institutional review boards of the University of California San Francisco and the University of California Berkeley.

### Participants

Patients presenting to the health centre with symptoms suggestive of malaria and a positive screening thick blood smear were consecutively referred to study physicians for further assessment. Patients were enrolled if they fulfilled the following selection criteria: 1) age one to ten years; 2) history of fever in the previous 24 hours or axillary temperature >37.5 °C; 3) no history of serious side effects to study medications; 4) no evidence of a concomitant febrile illness; 5) provision of informed consent by a parent or guardian; 6) no danger signs or evidence of severe malaria; and 7) P. falciparum mono-infection with parasite density 2,000–200,000/μl of blood. Because laboratory results were generally not available until the following day, a patient could be excluded after randomization. Patients were also excluded after randomization if they repeatedly vomited their first dose of study medications.

### Procedures

At enrollment, we asked children and their parents or guardians about prior antimalarial therapy, use of other medications, and presence of common symptoms. Axillary temperature and weight were measured and a physical examination was performed. A brief neurological assessment, consisting of simple clinical tests for hearing and fine finger dexterity (ability to pick up a small object) was undertaken. We also obtained blood by fingerprick for thick and thin blood smears, hemoglobin assessment, and to store on filter paper for molecular analysis.

Patients were asked to return for follow-up on days 1, 2, 3, 7, 14, 21, and 28, and any other day that they felt ill. Follow-up evaluation consisted of a standardised history and physical examination, including neurological assessment on days 7, 14, and 28. We obtained blood by fingerprick for thick blood smears and storage on filter paper on all follow-up days (except day 1). Hemoglobin measurement was repeated on day 28, or the day of recurrent symptomatic malaria. If patients did not return for follow-up, we visited them at home.

Blood smears were stained with 2% Giemsa for 30 min. Parasite densities were determined from thick blood smears by counting the number of asexual parasites per 200 white blood cells (or per 500, if the count was less than 10 parasites/200 white blood cells), assuming a white blood cell count of 8,000/μl. A smear was considered negative if no parasites were seen after review of 100 high-powered fields. We also assessed gametocytemia from thick blood smears. Thin blood smears were reviewed for non-falciparum infections. A second microscopist, who was unaware of the results of the first reading, re-read all slides. A third microscopist unaware of the first two readings resolved discrepant slides. Hemoglobin measurements were made using a portable spectrophotometer (HemoCue, Anglholm, Sweden).

### Interventions

On day 0, patients were randomly assigned to receive amodiaquine + artesunate or artemether–lumefantrine. A nurse administered study medications according to weight-based guidelines for administration of fractions of tablets (amodiaquine and artesunate) and the manufacturer's recommendations (artemether–lumefantrine). We administered all drugs orally as follows: amodiaquine (Camoquin, Parke-Davis, Pfizer, New York, New York, United States of America), 200 mg tablets, 25 mg/kg per treatment) 10 mg/kg on days 0 and 1, and 5 mg/kg on day 2; and artesunate (Arsumax, Sanofi-Aventis, Paris France), 50 mg tablets, 12 mg/kg per treatment, 4 mg/kg once daily for three days; artemether–lumefantrine (Coartem, Novartis, Basel, Switzerland), 20 mg artemether/120 mg lumefantrine tablets, 3-d six-dose regimen administered according to weight, as one [10–14 kg], two [15–24 kg], three [25–34 kg], or four [≥35 kg] tablets given twice daily for three days. Participants in the amodiaquine + artesunate group also received placebo tablets administered in the evening over three days, dosed similarly to weight-based guidelines for artemether–lumefantrine. Study medications were administered with water. Although participants were encouraged to resume normal food intake, no food was provided with the medications.

All treatment was directly observed. Participants were given the option either to wait at the clinic for the evening dose (lunch was provided) or to leave the clinic and return in the evening (transport was provided). After each dose, children were observed for 30 min and the dose was readministered if vomiting occurred. Children who repeatedly vomited their first dose of study medication were excluded from the study and referred for further management. We provided all patients with a 3-day supply of paracetamol for treatment of febrile symptoms. Those with a concentration of hemoglobin of less than 10.0 g/dL were treated according to Integrated Management of Childhood Illness guidelines with ferrous sulfate for 14 days and given antihelminthic treatment if appropriate.

### Objectives

The objectives of the study were to compare the efficacy and safety of amodiaquine + artesunate and artemether–lumefantrine for the treatment of uncomplicated falciparum malaria in Uganda.

### Outcomes—Efficacy

The primary efficacy outcomes were the 28-d risks of recurrent symptomatic malaria (early treatment failure and late clinical failure) and recurrent parasitemia (early treatment failure, late clinical failure, late parasitological failure), unadjusted and adjusted by genotyping. Secondary efficacy outcomes included risk of fever and parasitemia during the first three days of follow-up, change in mean hemoglobin from day 0 to day 28, or day of repeat therapy, and risk of gametocytemia during follow-up in participants lacking gametocytes at enrollment.

Treatment outcomes were classified according to 2003 WHO guidelines as early treatment failure (ETF; danger signs or complicated malaria or failure to adequately respond to therapy days 0–3), late clinical failure (LCF; danger signs or complicated malaria or fever and parasitemia on days 4–28 without previously meeting criteria for ETF), late parasitological failure (LPF; asymptomatic parasitemia day 28 without previously meeting criteria for ETF or LCF), and adequate clinical and parasitological response (ACPR; absence of parasitemia on day 28 without previously meeting criteria for ETF, LCF, or LPF) [12]. Patients classified as treatment failures were treated with quinine (10 mg/kg three times daily for seven days). Patients were excluded after enrollment if any of the following occurred: 1) use of antimalarial drugs outside of the study protocol; 2) parasitemia in the presence of a concomitant febrile illness; 3) withdrawal of consent; 4) loss to follow-up; 5) protocol violation; and 6) death due to a non-malaria illness.

Molecular genotyping techniques were used to distinguish recrudescent from new infections for all patients failing therapy after day 3. Briefly, filter paper blood samples collected on the day of enrollment and on the day of failure (LCF or LPF) were analyzed for polymorphisms in merozoite surface protein-1 (MSP-1) and −2 (MSP-2) using nested-PCR as previously described [13]. First, MSP-2 genotyping patterns on the day of failure were compared with those at treatment initiation using GelCompar II software for all paired samples (Applied Maths, Sint-Martens-Latem, Belgium). If all of the MSP-2 alleles present on the day of failure were present at the time of treatment initiation, genotyping was repeated using MSP-1. An outcome was defined as recrudescence if all MSP-1 and MSP-2 alleles present at the time of failure were present at the time of treatment initiation, and defined as a new infection otherwise.

### Outcomes—Safety

Secondary safety outcomes included risk of serious adverse events, and risk of events of moderate or greater severity. At each follow-up visit, patients were assessed for any new or worsening event. An adverse event was defined as any untoward medical occurrence, irrespective of its suspected relationship to the study medications (Guidance for Industry Good Clinical Practice: Consolidated Guidance [ICH E6], April 1996). All events were graded by severity (none, mild, moderate, severe, life-threatening) and relationship to study treatment (none, unlikely, possible, probable, or definite) using guidelines from the World Health Organization (Toxicity Grading Scale for Determining the Severity of Adverse Events) and the National Institutes of Health, Division of Microbiology and Infectious Diseases (Pediatric Toxicity Tables, May 2001). A serious adverse event was defined as any adverse experience that resulted in death, life-threatening experience, inpatient hospitalization, persistent or significant disability or incapacity, or specific medical or surgical intervention to prevent serious outcome.

### Sample Size

We calculated sample size to test the hypothesis that treatment with artemether–lumefantrine would decrease the risk of recurrent symptomatic malaria by 15% at 28 days compared with amodiaquine + artesunate. The risk over 28 days of repeat therapy for symptomatic malaria (unadjusted by genotyping) with amodiaquine + artesunate was estimated as 50% based on previous data [10]. Using this estimate, we calculated that 180 patients would need to be enrolled in each treatment arm (200 to allow for 10% loss to follow-up) to detect a 15% risk difference between the treatment groups with a two-sided type I error of 0.05 and 80% power. The sample size calculations for this study were performed online using the following Web site: http://statpages.org/proppowr.html.

### Randomization Procedures—Sequence Generation, Allocation Concealment, Implementation

An off-site investigator prepared computer-generated age-stratified randomization codes for two age groups (12–59 mo and 5–10 y) which were provided to a study nurse responsible for treatment allocation. The randomization list was secured in a locked cabinet accessible only by the study nurse. Participants were enrolled by the study physicians, and treatments were assigned and administered by the study nurse.

### Blinding

Only the study nurse was aware of treatment assignments. All other study personnel, including the study physicians and laboratory personnel involved in assessing outcomes, were blinded to the treatment assignments. Patients were not informed of their treatment regimen.

### Statistical Methods

Efficacy data were evaluated using a per-protocol analysis of patients with defined treatment outcomes. Analysis was performed using STATA version 8.0 (STATA, College Station, Texas, United States of America) statistical software program. Pair-wise comparisons of treatment efficacy were made using risk differences with exact 95% confidence intervals. Risks of recrudescence were estimated using the Kaplan–Meier product limit formula with censoring for new infections. Secondary outcomes included the presence of fever on days 1–3, parasitemia on days 2 and 3, change in hemoglobin level between the day of enrollment and the last day of follow-up, presence of gametocytes during any follow-up day, and the incidence of adverse events. Other categorical variables were compared using Chi-squared or Fisher's exact test and continuous variables were compared using the independent samples *t*-test. The non-parametric Wilcoxon rank sum test was used to analyze continuous data with a skewed distribution. All reported *p*-values are two sided without adjustment for multiple testing and were considered statistically significant if <0.05.

## RESULTS

### Participant Flow

**Figure 1:**
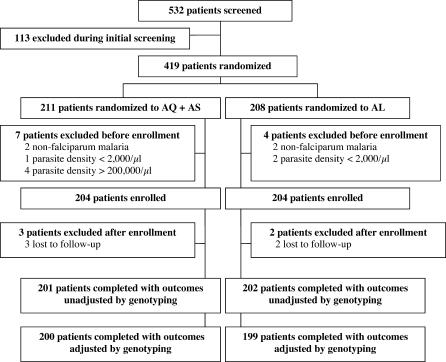
Trial Profile AQ + AS, amodiaquine + artesunate; AL, artemether–lumefantrine.

Of 532 patients screened, 419 were randomized to treatment, and 408 were enrolled in the study (Figure 1). Primary efficacy outcomes, unadjusted and adjusted by genotyping, were available for 403 (99%) and 399 (98%) enrolled participants, respectively.

### Recruitment

The study was conducted between December 2004 and July 2005.

### Baseline Data

Among patients with efficacy outcomes, there was no difference in baseline characteristics between the two treatment groups (Table 1).

### Numbers Analyzed

All 403 participants with unadjusted efficacy outcomes were included in the per-protocol analysis (Table 2).

### Outcomes and Estimation

#### Primary efficacy outcomes.

The risk of recurrent symptomatic malaria (unadjusted, including ETF and LCF) was significantly lower for participants treated with artemether–lumefantrine than for those treated with amodiaquine + artesunate (27% versus 42%, risk difference 15%, 95% CI 5.9%–24.2%). Similar results were seen for the risk of recurrent parasitemia (unadjusted, including ETF, LCF, and LPF, Table 2). The difference in risk of symptomatic malaria and parasitemia was nearly all due to the risk of LCF (26% artemether–lumefantrine versus 42% amodiaquine + artesunate, risk difference (16%, 95% CI 6.4%–24.7%); the risk of ETF and LPF was similar between the treatment groups.

Genotyping revealed that nearly all episodes of recurrent malaria were due to new infections (Table 2). In the artemether–lumefantrine group, one participant, aged 1.2 years, experienced a convulsion on day 0 associated with fever, following treatment with study medications, and was classified as an ETF. The parasite density was 80,760/μl on day 0, but had decreased to 2,440/μl on day 1. A second participant in this treatment group, aged 2.3 years, responded initially to treatment with artemether–lumefantrine, but presented on day 27 with fever and a parasite density of 600/μl, and after genotyping was classified as a recrudescent LCF.

**Figure 2:**
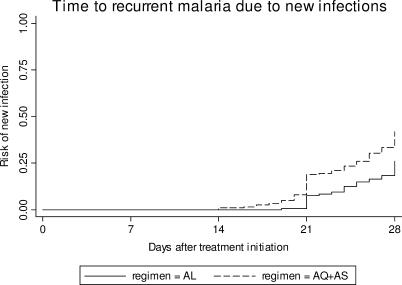
Day of Recurrent Malaria due to New Infection Stratified by Treatment Group

The time to recurrent malaria was significantly shorter in participants treated with amodiaquine + artesunate compared to those treated with artemether–lumefantrine (Figure 2). In patients treated with amodiaquine + artesunate, recurrent malaria was first identified on day 14, with 16 of 84 new infections identified before day 21 (*n* = 84, median 23.5 days, range 14–28 days). In contrast, in the artemether–lumefantrine group only one new infection occurred before day 21 (*n* = 52, median 25 days, range 19–28 days, *p*-value = 0.05).

#### Secondary efficacy outcomes.

Both treatments produced rapid clearance of parasitemia (Table 3). Although there was no difference in parasite clearance between the two treatment groups, patients treated with amodiaquine + artesunate experienced more rapid resolution of fever on days 1 and 2 than those treated with artemether–lumefantrine. There was no difference in mean change in hemoglobin between the two groups. The proportion of patients with any gametocytes during follow-up was significantly lower in the artemether–lumefantrine group (20% artemether–lumefantrine versus 31% amodiaquine + artesunate). Similar results were found for patients with newly emerging gametocytes during follow-up (5% artemether–lumefantrine versus 15% amodiaquine + artesunate).

### Ancillary Analyses

#### Seasonal variation.

**Figure 3:**
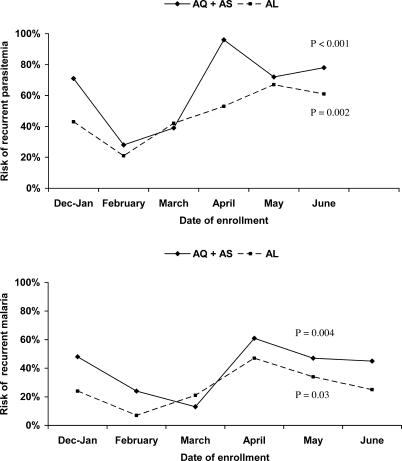
Risk of Recurrent Parasitemia and Recurrent Malaria Stratified by Date of Enrollment The peak in risks corresponds to the period following the rainy season. The *p*-values are for differences in risks across the different time periods for each regimen.

The risk of recurrent parasitemia and recurrent symptomatic malaria in this study varied over time (Figure 3). The risk for both outcomes was lowest between February and March, during the dry season, and peaked from April to June, following the rainy season. Remarkably, during the time of peak transmission, the risk of recurrent parasitemia over 28 days was more than 50% and the risk of recurrent symptomatic malaria was more than 30% for both treatment arms.

### Adverse Events

Artemether–lumefantrine and artesunate + amodiaquine were both well-tolerated. Overall, 261 (65%) of participants experienced any adverse event of moderate or greater severity, and there was no difference between the two treatment groups (Table 3). No abnormalities in hearing or fine finger dexterity were detected. A more detailed accounting of these results will be reported separately. Serious adverse events occurred in two participants. One child treated with amodiaquine + artesunate developed pneumonia on day 27, requiring hospitalization, but the event was judged to be unrelated to study medications. A second participant, treated with artemether–lumefantrine, experienced a convulsion on day 0 (classified as an ETF as described above), which was judged to be unlikely to be related to the study medication.

## DISCUSSION

### Interpretation

In this randomized clinical trial, amodiaquine + artesunate and artemether–lumefantrine were both highly efficacious for treatment of uncomplicated malaria in Tororo, an area with a very high level of transmission of malaria. No recrudescences occurred in patients treated with amodiaquine + artesunate and only two occurred in those treated with artemether–lumefantrine, although additional recrudescences might have been detected if follow-up had been extended to at least 42 days. The performance of artemether–lumefantrine was consistent with results from other recent studies from East Africa [14–16] and prior studies from Asia [17,18], suggesting that this combination may be highly effective in areas with considerable resistance to other antimalarial drugs. This is welcome news for Uganda, where artemether–lumefantrine was recently adopted as the new first-line therapy for uncomplicated malaria, and amodiaquine + artesunate has been recommended as an alternative.

**Table 1:**
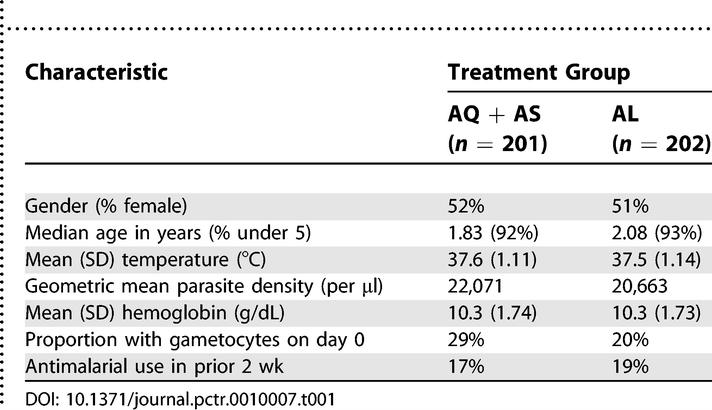
Baseline Characteristics of Patients Completing the Study

In this study, despite the excellent performance of amodiaquine + artesunate and artemether–lumefantrine in terms of short-term efficacy, the proportion of patients experiencing recurrent parasitemia within one month was substantial in both treatment groups. Two-thirds of patients treated with amodiaquine + artesunate, and half of those treated with artemether–lumefantrine, were parasitemic during 28 days of follow-up. Nearly all recurrent parasitemias were characterized as new infections. Nonetheless, even with the more efficacious regimen (artemether–lumefantrine), over a quarter of patients had recurrent illness, demonstrating the importance of reinfection in areas of very high malaria transmission. This result highlights the need to re-evaluate the approach to treatment of recurrent episodes of malaria following initial ACT therapy. In our study, participants received quinine as second-line treatment. However it is unclear whether quinine, the same ACT, or a different ACT would be the optimal treatment. Given that nearly all recurrent parasitemias were due to new infections, it may be reasonable to retreat with the same ACT regimen, rather than with quinine. Our results also indicate that integration of approaches to control malaria is essential. In an area of South Africa with lower-intensity transmission, the combination of vector control measures and provision of artemether–lumefantrine dramatically decreased the malaria burden [19]. In areas of high transmission, even highly efficacious antimalarial treatments may need to be given repeatedly, which has obvious cost implications, and may significantly impact on the safety of drug regimens.

We found that artemether–lumefantrine was superior to amodiaquine + artesunate for preventing new infections, consistent with recent results from Zanzibar [15]. Because both artemisinin derivatives are rapidly eliminated, our results suggest that lumefantrine has a greater post-treatment prophylactic effect than amodiaquine, perhaps due to the waning of amodiaquine efficacy due to drug resistance [20]. Delayed drug clearance may prevent new infection with drug sensitive parasites, but is likely to contribute to the selection of drug resistance, and may impact on long-term efficacy [21]. In Zanzibar, artemether–lumefantrine was associated with post-treatment selection of the *pfmdr1* 86N allele, which may play a role in lumefantrine resistance [15,22]. As ACT use becomes widespread in areas with high levels of malaria transmission, it will be important to monitor closely for the selection of parasites that are resistant to artemisinin partner drugs. The benefits of a regimen that prevents recurrent infection, particularly in areas of high transmission, could be substantial. However, these benefits will need to be weighed against the potential for driving drug resistance.

**Table 2:**
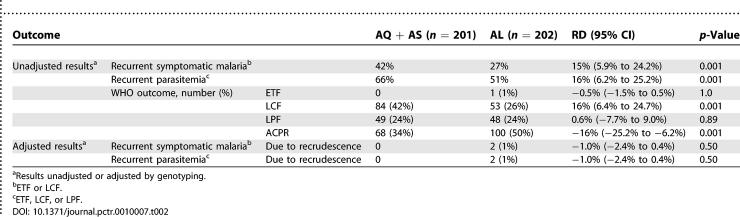
Primary Treatment Outcomes

### Generalizability

The risk of recurrent parasitemia and symptomatic malaria had significant seasonal variation. Although malaria transmission occurs year-round in Tororo, the difference in transmission between dry and wet seasons had a substantial impact on risk of new infection, and therefore on the need for retreatment. The seasonal variation is important to recognize when comparing results of drug efficacy studies conducted at the same site. More broadly, these results highlight the importance of the level of transmission in determining risks of recurrent malaria after therapy. In a prior drug efficacy study conducted at the same site between September 2003 and March 2004, the unadjusted risk of recurrent infection at 28 days was 74% for amodiaquine + artesunate versus 59% for amodiaquine + sulfadoxine-pyrimethamine, with 12% versus 18% risk of recrudescence, respectively [10]. For amodiaquine + artesunate, the difference between the past results and those reported here is likely due to seasonal and/or annual variation (impacting on risk of recurrent parasitemia), as well as slight differences in genotyping techniques (impacting on risk of recrudescence). In the prior study, genotyping was performed by analyzing for polymorphisms in MSP-2 only. In this study, the genotyping technique was refined by analyzing all samples initially identified as recrudescent by MSP-2 analysis for polymorphisms in MSP-1. Using this refined technique, the majority of samples initially classified as recrudescent were reclassified as new infections. Amodiaquine + sulfadoxine-pyrimethamine was not included in this study and thus cannot be directly compared to artemether–lumefantrine. However, the risks of recurrent parasitemia for amodiaquine + artesunate versus amodiaquine + sulfadoxine–pyrimethamine in the prior study (74% versus 59%, risk difference 15%, 95% CI 5%–25%) were similar to those for amodiaquine + artesunate versus artemether–lumefantrine in the current study (66% versus 51%, risk difference 16%, 95% CI 6%–25%).

**Table 3:**
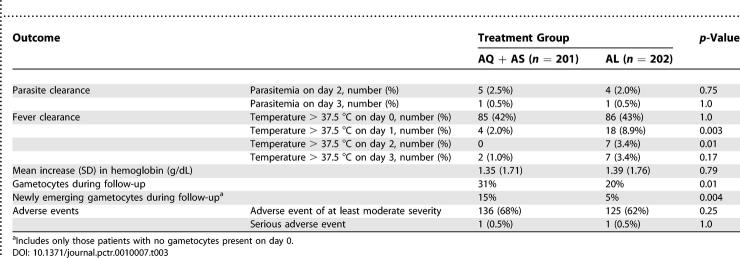
Secondary Outcomes

### Overall Evidence

A systematic review of the six-dose regimen of artemether–lumefantrine published in 2005 identified nine randomized controlled trials, including four studies conducted in Africa (in Burundi, The Gambia, Tanzania, and Uganda) [14,16,23–25]. The reviewers concluded that the six-dose regimen of artemether–lumefantrine appeared to be more effective than regimens that did not contain an artemisinin. In the two studies which compared artemether–lumefantrine to amodiaquine + artesunate (an effectiveness study in Tanzania and an efficacy trial in Burundi), artemether–lumefantrine resulted in fewer failures by day 28 in Tanzania, and fewer parasitological failures by day 14 in both Tanzania and Burundi [16,24]. The study from Uganda compared supervised versus unsupervised administration of artemether–lumefantrine and found that both methods were highly effective (98% genotyping-adjusted cure at day 28 with both methods) [14]. A more recent study comparing artemether–lumefantrine to amodiaquine + artesunate in Zanzibar found that both regimens were highly efficacious after 42-d follow-up (genotyping-adjusted cure rates 94% and 91%, respectively, when uncertain PCR results were defined as re-infections, *p* = 0.115), and that artemether–lumefantrine provided greater protection against re-infection compared with amodiaquine + artesunate [15].

Our study adds to the evidence base on the comparative efficacy of ACTs in Africa. The results from Tororo support the findings of the Zanzibar trial, although the overall risk of recurrent malaria at 28 days was substantially higher in our study (66% amodiaquine + artesunate versus 51% artemether–lumefantrine) than in Zanzibar (28% amodiaquine + artesunate versus 7% artemether–lumefantrine, likely reflecting the greater level of malaria transmission at our study site [15]. We found that amodiaquine + artesunate and artemether–lumefantrine were both highly efficacious, but each regimen has limitations. The lifespan of amodiaquine + artesunate may be limited by resistance to amodiaquine, and the current lack of co-formulation of this regimen may reduce adherence, although a co-formulated combination may be available soon. Both regimens are much more expensive than older antimalarial drugs, and their widespread use in Africa has been challenged by limited availability. For both ACT regimens, the high rate of re-infection and the implications of frequent retreatment is a major concern. Artemisinin combination therapy offers an important step forward for the treatment of malaria in Africa, but continued research into effective, safe, and affordable antimalarial regimens, consideration of the comparative post-treatment effects of different therapies, and integration of treatment with preventive methods, will be necessary to establish effective and sustainable malaria control policies.

## SUPPORTING INFORMATION

CONSORT Checklist Click here for additional data file. (51 KB DOC).

Trial Protocol Click here for additional data file. (821 KB DOC).

## Abbreviations

ACPR: adequate clinical and parasitological response
ACT: artemisinin combination therapy
AL: artemether–lumefantrine
AQ+AS: amodiaquine + artesunate
ETF: early treatment failure
LCF: late clinical failure
LPF: late parasitological failure
MSP: merozoite surface protein
WHO: World Health Organization
