# Supplementary material for: Artemisinin Combination Therapies for Treatment of Uncomplicated Malaria in Uganda
Source: PLoS Clin Trials. 2006 May 19;1(1):e7. doi: 10.1371/journal.pctr.0010007 (PMC1488893; doi:10.1371/journal.pctr.0010007)
Supplement: Trial Protocol [file pctr.0010007.sd002.doc]

.

**COMPARISON OF AMODIAQUINE PLUS ARTESUNATE AND**

**ARTEMETHER-LUMEFANTRINE FOR TREATMENT OF**

**UNCOMPLICATED MALARIA IN UGANDA:**

**EVALUATION OF EFFICACY, SAFETY, AND TOLERABILITY**

**Protocol version 1.1**

**Uganda Malaria Surveillance Project drug efficacy studies**

**19 October 2004**

TABLE OF CONTENTS

[ABBREVIATIONS and ACRONYMS 4](#__RefHeading___Toc84321123)

[1.0 STUDY SUMMARY 5](#__RefHeading___Toc84321124)

[2.0 INTRODUCTION 6](#__RefHeading___Toc84321125)

[2.1 Background 6](#__RefHeading___Toc84321126)

[2.1.1 Revision of the antimalarial drug policy in Uganda 7](#__RefHeading___Toc84321127)

[2.2 Study drugs 7](#__RefHeading___Toc84321128)

[2.2.1 Combination antimalarial therapy 7](#__RefHeading___Toc84321129)

[2.2.2 Amodiaquine + artesunate (AQ+AS) 7](#__RefHeading___Toc84321130)

[2.2.3 Artemether plus lumefantrine (Coartemether) 8](#__RefHeading___Toc84321131)

[2.3 Rationale 8](#__RefHeading___Toc84321132)

[3.0 STUDY AIM 8](#__RefHeading___Toc84321133)

[4.0 STUDY DESIGN 9](#__RefHeading___Toc84321134)

[4.1 Overall study design 9](#__RefHeading___Toc84321135)

[4.2 Study outcome and assessment 9](#__RefHeading___Toc84321136)

[4.2.1 Classification of treatment outcome 9](#__RefHeading___Toc84321137)

[4.2.2 Primary outcome 9](#__RefHeading___Toc84321138)

[4.2.3 Secondary outcomes 9](#__RefHeading___Toc84321139)

[4.3 Randomization: 10](#__RefHeading___Toc84321140)

[5.0 PARTICIPANT SELECTION AND ENROLLMENT 10](#__RefHeading___Toc84321141)

[5.1 Study site 10](#__RefHeading___Toc84321142)

[5.2 Recruitment and screening process (Appendix 1) 10](#__RefHeading___Toc84321143)

[5.3 Selection criteria 10](#__RefHeading___Toc84321144)

[6.0 BASELINE EVALUATION AND TREATMENT ALLOCATION 11](#__RefHeading___Toc84321145)

[6.1 Baseline evaluation and procedures 11](#__RefHeading___Toc84321146)

[6.2 Treatment group assignment 11](#__RefHeading___Toc84321147)

[6.3 Treatment allocation 11](#__RefHeading___Toc84321148)

[6.4 Study intervention 12](#__RefHeading___Toc84321149)

[6.4.1 Dosing of study medications 12](#__RefHeading___Toc84321150)

[6.5 Treatment administration and blinding 12](#__RefHeading___Toc84321151)

[6.5.1 Treatment administration at the clinic 12](#__RefHeading___Toc84321152)

[6.6 Additional medications 13](#__RefHeading___Toc84321153)

[7.0 FOLLOW-UP EVALUATION AND PROCEDURES 13](#__RefHeading___Toc84321154)

[7.1 Follow-up schedule 13](#__RefHeading___Toc84321155)

[Table 4. Follow-up Schedule 13](#__RefHeading___Toc84321156)

[7.2 Classification of treatment outcome and management of treatment failure 13](#__RefHeading___Toc84321157)

[7.3 Exclusion after enrollment and loss to follow-up 14](#__RefHeading___Toc84321158)

[7.4 Management of non-malarial illnesses 14](#__RefHeading___Toc84321159)

[8.0 ASSESSMENT FOR ADVERSE EVENTS 14](#__RefHeading___Toc84321160)

[8.1 Definitions 14](#__RefHeading___Toc84321161)

[8.2 Identification of adverse events 14](#__RefHeading___Toc84321162)

[8.3 Reporting of adverse events 15](#__RefHeading___Toc84321163)

[8.4 Reporting of serious adverse events 15](#__RefHeading___Toc84321164)

[9.0 STATISTICAL CONSIDERATIONS 15](#__RefHeading___Toc84321165)

[9.1 Sample size calculations 15](#__RefHeading___Toc84321166)

[9.2 Analytical plan 15](#__RefHeading___Toc84321167)

[10.0 DATA COLLECTION AND MANAGEMENT 16](#__RefHeading___Toc84321168)

[10.1 Data management 16](#__RefHeading___Toc84321169)

[10.2 Data quality assurance and monitoring 16](#__RefHeading___Toc84321170)

[10.3 Records 16](#__RefHeading___Toc84321171)

[11.0 LABORATORY PROCEDURES 16](#__RefHeading___Toc84321172)

[11.1 Blood smears 16](#__RefHeading___Toc84321173)

[11.2 Haemoglobin measurement 17](#__RefHeading___Toc84321174)

[11.3 Molecular studies 17](#__RefHeading___Toc84321175)

[12.0 PROTECTION OF HUMAN SUBJECTS 17](#__RefHeading___Toc84321176)

[12.1 Institutional Review Board (IRB) review and informed consent 17](#__RefHeading___Toc84321177)

[12.2 Risks and discomforts 17](#__RefHeading___Toc84321178)

[12.2.1 Privacy 17](#__RefHeading___Toc84321179)

[12.2.2 Risks of randomization 17](#__RefHeading___Toc84321180)

[12.2.3 Fingerprick blood draws 17](#__RefHeading___Toc84321181)

[12.2.4 Risk of amodiaquine 17](#__RefHeading___Toc84321182)

[12.2.5 Risk of artemisinins 18](#__RefHeading___Toc84321183)

[12.2.6 Artemether-Lumefantrine (Coartemether) 19](#__RefHeading___Toc84321184)

[12.2.7 Risk of Quinine 20](#__RefHeading___Toc84321185)

[12.3 Compensation 20](#__RefHeading___Toc84321186)

[12.4 Consent procedures 20](#__RefHeading___Toc84321187)

[12.5 Alternatives 20](#__RefHeading___Toc84321188)

[12.6 Confidentiality of records 20](#__RefHeading___Toc84321189)

[13.0 TIMETABLE 21](#__RefHeading___Toc84321190)

[14.0 REFERENCES 22](#__RefHeading___Toc84321191)

[APPENDIX 1. PARTICIPANT SELECTION AND ENROLLMENT 25](#__RefHeading___Toc84321192)

[Appendix 2: Patient Screening Form 26](#__RefHeading___Toc84321193)

[APPENDIX 3. UMSP ENROLLMENT FORM. 27](#__RefHeading___Toc84321194)

[APPENDIX 4. Critical steps 28](#__RefHeading___Toc84321195)

[Appendix 5. Informed Consent 29](#__RefHeading___Toc84321196)

[Appendix 6a. Weight-based administration of study medications for AQ+AS group 38](#__RefHeading___Toc84321197)

[Appendix 6b. Weight-based administration of study medications for coartemether group 39](#__RefHeading___Toc84321198)

[APPENDIX 7. CLASSIFICATION OF RESPONSE TO TREATMENT 40](#__RefHeading___Toc84321199)

[Appendix 8. Criteria for Severe Malaria/Danger Signs 41](#__RefHeading___Toc84321200)

[Appendix 9. Guidelines for Grading Patient Symptoms, signs and laboratory findings. 42](#__RefHeading___Toc84321201)

[Table A. Guidelines for grading patient symptoms. 42](#__RefHeading___Toc84321202)

[Table B. Guidelines for Physical Examination 43](#__RefHeading___Toc84321203)

[Table C. Grading Physical Examination Findings 44](#__RefHeading___Toc84321204)

[TABLE D. Guidelines for Grading of Laboratory Abnormalities 45](#__RefHeading___Toc84321205)

[Appendix 10. Adverse Event - Follow-up Report 46](#__RefHeading___Toc84321206)

[Appendix 11. Serious Adverse Event Form – Initial report. 47](#__RefHeading___Toc84321207)

[Appendix 12. Clinical Record forms 53](#__RefHeading___Toc84321208)

# ABBREVIATIONS and ACRONYMS

UMSP: Uganda Malaria Surveillance Project

MoH: Ugandan Ministry of Health

EANMAT: East African Network for Monitoring Antimalarial Therapy

CQ: Chloroquine

AQ: Amodiaquine

AS: Artesunate

SP: Sulphadoxine pyrimethamene

US: United States

UCSF: University of California San Francisco

WHO: World Health Organisation

ACPR: Adequate clinical and parasitological response

ETF: Early treatment failure

LCF: Late clinical failure

LPF: Late parasitological failure

IMCI: Integrated Management of Childhood Illness

Hb: Haemoglobin

DHPS: Dihydropterate synthetase

DHFR: Dihydrofolate reductase

IRB: Institutional Review Board

DNA: Deoxyribonucleic acid

GCP: Good Clinical Practice.

# 1.0 STUDY SUMMARY

| **Title** | **Comparison of amodiaquine + artesunate and artemether + lumefantrine for treatment of uncomplicated malaria in Uganda: evaluation of efficacy, safety, and tolerability** |
| --- | --- |
| **Description** | Randomised, single-blinded trial |
| **Participants and sample size** | Ugandans aged > 6 months recruited from 4 study sites  Total sample size 1600 subjects, 400 per site  (2 treatment arms at each site with 200 subjects per treatment arm) |
| **Clinical Sites** | The study will be conducted at 4 of Uganda Malaria Surveillance Project (UMSP) sentinel sites namely, Tororo, Kanungu, Mubende and Arua. |
| **Selection Criteria** | 1. Not previously enrolled in this study 2. Age **1 – 10 years** 3. Weight > 10 kg 4. Fever (> 37.5ºC axillary) or history of fever in the previous 24 hours 5. Absence of any history of serious side effects to study medications 6. No evidence of a concomitant febrile illness 7. Provision of informed consent and agreement to follow-up for 28 days 8. No evidence of severe malaria or danger signs 9. Absence of repeated vomiting of study medications on day 0 10. *P. falciparum* mono-infection 11. Parasite density > 2000/ul and < 200,000/ul |
| **Study intervention** | Subjects will be randomized to treatment with amodiaquine + artesunate (AQ+AS) or artemether + lumefantrine (coartemether). Subjects in the AQ+AS arm will also receive placebo tablets to ensure that the number of doses received is identical in the two treatment groups. Subjects requiring repeat therapy (second-line therapy given for symptomatic malaria) will receive quinine. |
| **Follow-up** | Subjects will be followed for 28 days and will be asked to return for follow-up assessment on days 1, 2, 3, 7, 14, 21, 28, and any unscheduled day that they feel ill. |
| **Primary outcome** | Risk of clinical rescue therapy (second-line therapy given for symptomatic malaria) |
| **Secondary outcomes** | 1. Risk of clinical treatment failure (due to recrudescence) 2. Risk of parasitological rescue therapy 3. Risk of parasitological treatment failure 4. Risk of fever on days 1-3 5. Risk of parasitemia on days 2 and 3 6. Change in mean haemoglobin between days 0 and 28, or day of treatment failure 7. Proportion of patients with gametocytaemia during follow-up 8. Risk of serious adverse events during follow-up 9. Risk of adverse events of moderate or greater severity, at least possibly related to the study medications, excluding patients requiring rescue therapy |

# 2.0 INTRODUCTION

## 2.1 Background

Malaria remains one of the most serious global health problems and a leading cause of morbidity and mortality in Uganda. Appropriate case management, focusing on prompt treatment with effective antimalarial drugs, is the foundation of malaria control throughout sub-Saharan Africa. For decades, chloroquine (CQ) was the mainstay of antimalarial therapy, but the emergence of *P. falciparum* resistance to CQ has challenged control efforts.[1] The spread of CQ resistance has coincided with increased malaria-related morbidity and mortality in Africa, highlighting the urgent need to change antimalarial treatment policy in the face of rising CQ resistance.[2] However, the optimal replacement to CQ has not been clear and available alternatives are limited. In 2000, the Ugandan Ministry of Health (MoH) re-evaluated the national antimalarial drug policy in response to expanding CQ resistance. The combination of CQ plus sulfadoxine-pyrimethamine (CQ+SP) was chosen to replace CQ as first-line treatment for uncomplicated malaria. However, at the time that this decision was made, the efficacy and safety of the CQ+SP combination had not been evaluated in Uganda.

In 2002, the Uganda Malaria Surveillance Project (UMSP), a collaboration involving the MoH and academic partners, was formed to collect data on antimalarial therapies to aid the MoH in drug policy decision-making. A multi-site study was conducted at 7 sentinel sites in Uganda in 2002-2004 to compare the efficacy and safety of CQ+SP to potential alternatives for first-line therapy, including amodiaquine + SP (AQ+SP), and amodiaquine + artesunate (AQ+AS). Data from this study and a trial comparing the same combination regimens in Kampala are presented below in Table 1.(UMSP, unpublished data)[3] In summary, the risk of treatment failure with CQ+SP was unacceptably high at all sites. Treatment with AQ+SP or AQ+AS greatly reduced the risk of rescue therapy (for recrudescence or new infection) and the risk of true treatment failure compared to CQ+SP. Comparing the AQ-containing regimens, the risk of true treatment failure was lower with AQ+AS, but this was offset by a higher risk of new infection. Overall, AQ+SP lowered the risk of rescue therapy, especially at the sites with the highest transmission intensity.

**Table 1. Ugandan drug efficacy studies (2002-04): 28-day treatment outcomes in children less than 5 years**

| **Site** | **CQ+SP** | **AQ+SP** | **AQ+AS** |
| --- | --- | --- | --- |
| **Risk of Clinical Rescue Therapy*** | | | |
| Kanungu | 86% | 53% | - |
| Mubende | 70% | 44% | - |
| Kyenjojo | 70% | 35% | - |
| Kampala | 45% | 20% | 16% |
| Jinja | 57% | 19% | 19% |
| Tororo | 75% | 45% | 55% |
| Apac | 32% | 17% | 28% |
| **Risk of Clinical Treatment Failure†** | | | |
| Kanungu | 73% | 37% | - |
| Mubende | 42% | 13% | - |
| Kyenjojo | 58% | 22% | - |
| Kampala | 39% | 15% | 4% |
| Jinja | 49% | 14% | 6% |
| Tororo | 34% | 19% | 12% |
| Apac | 10% | 2% | 6% |

* Rescue therapy defined as quinine treatment given to a study subject when a treatment failure was identified based on results unadjusted by genotyping, and includes all early and late treatment failures

† Clinical treatment failure includes all early treatment failures and late treatment failures due to recrudescence after adjustment by genotyping (censoring new infections).

## 2.1.1 Revision of the antimalarial drug policy in Uganda

Available data from recently conducted efficacy studies were evaluated at a national consensus meeting in May 2004 to review Uganda's national antimalarial drug policy. Given substantial evidence that CQ+SP was failing, a decision was made to abandon this as the first-line regimen. Despite limited data on the comparative efficacy, safety, and cost-effectiveness of alternative regimens, there was urgent need to select a replacement regimen for first-line treatment, and only artemisinin-based combination therapies (ACTs) are being considered for first-line treatment. Although AQ+AS is an option, concern about the safety and tolerability of AQ, in addition to AQ resistance, may limit support for this combination. Coartemether, a co-formulated regimen that combines artemether, an artemisinin derivative, with the novel agent lumefantrine, is the likely replacement for first-line therapy, although data on this combination are very limited in Uganda. To facilitate rational drug policy decision-making, the investigation of alternative antimalarial regimens is essential.

## 2.2 Study drugs

### 2.2.1 Combination antimalarial therapy

Combination therapy has become the standard for the treatment of many infectious diseases. The advantages of combination therapy are improved efficacy and decreased selection of resistant organisms. Combination antimalarial therapy has already become standard in Southeast Asia, including Thailand where artesunate plus mefloquine is the standard regimen for falciparum malaria in areas with high-level drug resistance [4, 5]. Use of combination therapy, particularly ACT has also been strongly advocated in Africa [6] However, despite the substantial international support for ACTs, obstacles to widespread use of these regimens exist, including safety, cost, and drug availability.

### 2.2.2 Amodiaquine + artesunate (AQ+AS)

**Amodiaquine**

Amodiaquine (AQ) is very similar in structure to chloroquine (CQ), and the two drugs probably share mechanisms of action. Despite concerns about cross-resistance with CQ, AQ generally retains activity for the treatment of uncomplicated malaria in settings where CQ treatment failure is very common [7]. Use of AQ has been limited since the 1980’s due to concerns over toxicity, primarily hepatotoxicity and blood dyscrasias in individuals receiving long-term therapy for chemoprophylaxis [7]. However, AQ has remained available and inexpensive in Africa, and it has been fairly widely used as a replacement for CQ. Recent studies have suggested that AQ is, indeed, efficacious and safe when used for the treatment of uncomplicated malaria, even in areas of high-level CQ resistance [7-11]. In a study from Kampala, efficacy with AQ monotherapy has been shown to be markedly superior compared to previous studies with CQ alone in the same target population (14-day treatment failure rate 7% vs. 47%) [11, 12]. Based on data from multiple studies showing limited toxicity and marked improvement in efficacy over CQ, AQ is now increasingly advocated for use in the treatment of uncomplicated malaria in Africa.

**Artemisinin derivatives**

Artemisinins are a recently developed class of antimalarials that are natural products or semi-synthetic compounds derived from the plant *Artemesia annua* [13]. The artemisinins offer very rapid and potent treatment for malaria, and they benefit from a lack of known drug resistance. However, artemisinin derivatives are currently expensive, and because of their short half-life ideally should be given for extended courses or in combination with other drugs. Artemisinin derivatives given at high doses produce a selective neurotoxicity in laboratory animals, but this toxicity has not been observed in humans, and the drugs appear to be safe and well-tolerated.[14] Evidence of reproductive toxicity in animals treated with artemisinins in early pregnancy is of perhaps greater concern, and has led the WHO to recommend avoiding the use of artemisinin compounds in the first trimester.[15]

**Amodiaquine plus artesunate (AQ+AS)**

In recent comparative studies conducted in Kampala, Jinja, Tororo and Apac districts in Uganda, AQ+AS was well-tolerated and showed superior efficacy over CQ+SP. Compared to AQ+SP, AQ+AS had a lower risk of clinical treatment failure but a higher risk of new infection (UMSP, unpublished data and Staedke, in press) [3]. A multi-center study evaluating AQ+AS in Kenya, Senegal, and Gabon, which included 470 treatments, showed that the regimen was well-tolerated and provided excellent efficacy for uncomplicated malaria [16]. Three countries in Africa (Burundi, Gabon, and Zanzibar) have selected the combination of AQ+AS as first-line therapy [17].

### 2.2.3 Artemether plus lumefantrine (Coartemether)

Coartemether (Coartem) is an oral preparation containing the artemisinin derivative, artemether, and lumefantrine (previously known as benflumetol). As with other artemisinins, artemether is characterized by rapid antimalarial action, however, recrudescence is frequent when artemether is provided as a single agent, unless given for at least 5-7 days [18, 19]. Lumefantrine also has a high cure rate, but parasite and fever clearance is slower than with artemether [20]. The fact that coartemether combines an artemisinin derivative with a novel agent in a fixed dose regimen is an important advantage of this therapy. It is currently the only co-formulated antimalarial regimen and is included on the WHO Essential Drugs List [21]. However, coartemether is administered twice daily (a total of 6 doses over 3 days), and should ideally be taken with fatty food or liquid to maximize absorption, raising concerns about adherence.[22] In addition, the currently recommended 6 dose regimen has been little studied in Africa.[22] Coartemether has been selected as first-line therapy in South Africa (Kwazulu Natal), Zambia, and Comoros, and as second-line therapy for treatment of first-line failures in Cote d'Ivoire, Gabon, Mozambique, Senegal, and Zanzibar. [23]

## 2.3 Rationale

ACTs have been strongly advocated for use in Africa, but data on these regimens are currently limited. In recent UMSP studies, AQ+AS was safe and efficacious. However, compared to AQ+SP, the risk of recrudescence with AQ+AS was significantly lower at only one site (Kampala), while the risk of new infection with this regimen was significantly higher in 2 of 4 sites (Apac and Tororo) (UMSP, unpublished data and Staedke, in press) [3]. Coartem has been provisionally chosen as the replacement to CQ+SP as first-line therapy, but there is little data on the efficacy and safety of this regimen in Uganda, and its substantial cost (US$ 2.40 per adult treatment vs. US$ 1.30 for AQ+AS) [24] remains a significant obstacle. We are proposing to compare the efficacy, safety and tolerability of AQ+AS and Coartem for the treatment of uncomplicated falciparum malaria in Uganda. The data that is collected in this study will be made directly available to the Ugandan MoH to assist in drug policy decision-making.

# 3.0 STUDY AIM

To compare the efficacy, safety, and tolerability of AQ+AS and coartemether for the treatment of uncomplicated falciparum malaria in Uganda.

# 4.0 STUDY DESIGN

## 4.1 Overall study design

The study will be a randomised, single-blinded trial designed according to 2003 World Health Organization (WHO) guidelines for assessment of therapeutic efficacy of antimalarial agents in areas of low, moderate and intense transmission [25] with slight modifications. The target population includes residents of the catchment areas of the four clinical sites in Uganda. The available population includes residents aged 6 months and older who present to the study clinics with symptoms suggestive of malaria and who have a positive screening thick blood smear. Subjects who meet the selection criteria will be randomized to treatment with one of the two study regimens and will be followed for 28 days. Repeat evaluations will be performed on days 1, 2, 3, 7, 14, 21, and 28 (and any unscheduled day) and will include assessment for the occurrence of adverse events. Treatment efficacy outcomes will be assessed using WHO outcome classification criteria (Appendix 7).

## 4.2 Study outcome and assessment

### 4.2.1 Classification of treatment outcome

Response to treatment will be classified according the recently proposed WHO classification system, and will include adequate clinical and parasitological response (ACPR), early treatment failure (ETF), late clinical failure (LCF), and late parasitological failure (LPF), for purposes of data reporting (Appendix 3). [26] In the final analysis, treatment outcomes will be dichotomized based on the following definitions:

- Clinical rescue therapy = ETF + LCF
- Parasitological rescue therapy = ETF + LCF + LPF.

All ETFs will be considered true treatment failures. For all LCFs and LPFs, molecular genotyping will be used to distinguish recrudescence (true treatment failure) from new infection (see section 11.3).

- Clinical treatment failure = All ETFs + LCFs due to recrudescence
- Parasitological treatment failure = All ETFs + LCFs/LPFs due to recrudescence

### 4.2.2 Primary outcome

Primary outcome will be based on the risk of clinical rescue therapy. Pairwise comparisons between regimens will be made based on a per-protocol analysis.

### 4.2.3 Secondary outcomes

1. Risk of clinical treatment failure
2. Risk of parasitological rescue therapy
3. Risk parasitological treatment failure
4. Risk of fever during the first 3 days of follow-up: presence or absence of objective fever (axillary temperature > 37.5°C) or patient report of fever on days 1, 2, 3
5. Risk of parasitemia on follow-up days 2 and 3: proportion of positive vs. negative thick blood smears on day 2 and day 3
6. Change in mean haemoglobin from day 0 to 28 or day of repeat therapy
7. Proportion of subjects lacking gametocytes on day 0 with gametocytaemia on any follow-up day
8. Risk of serious adverse events: proportion of patients experiencing any serious adverse event in each treatment group during the 28-day follow-up period, excluding treatment failures
9. Risk of adverse events of moderate or greater severity, at least possibly related to the study medications, excluding treatment failures.

### 4.3 Randomization:

Computer generated randomization lists will be created for each of the 4 study sites by a member of the project who will not be directly involved in the conduct of the study. Sealed copies of the original randomization lists and documentation of the procedure used to generate the lists will be stored in the project administrative offices in Kampala. Prior to the onset of the study, sealed copies of the randomization lists will be distributed to the study nurse responsible for treatment allocation.

# 5.0 PARTICIPANT SELECTION AND ENROLLMENT

## 5.1 Study site

The study will be conducted at 4 of the Uganda Malaria Surveillance Project (UMSP) sentinel sites that were originally established in 1998 by the Ugandan Ministry of Health (MoH) in collaboration with the East African Network for Monitoring Antimalarial Treatment (EANMAT).

Table 2. UMSP sentinel sites

| **District** | **Health center** | **Region type** | **Catchment population** | **Transmission** |
| --- | --- | --- | --- | --- |
| Tororo | Nagongera | Rural | 26,010 | Holoendemic |
| Kanungu | Kihihi | Rural | 14,000 | Mesoendemic |
| Mubende | Kasambya | Rural | 40,000 | Hyperendemic |
| Arua | Chilio | Rural | 15,685 | Holoendemic |

## 5.2 Recruitment and screening process (Appendix 1)

Study subjects will be recruited from the OPD (outpatient department) of the sentinel health centre. Patients who present with symptoms suggestive of malaria (fever or history of recent fever) will be referred to the outpatient laboratory for a screening thick blood smear (using standard Giemsa staining). Thick blood slides will be read and counted by the laboratory technicians. The parasite density of positive screening thick blood smears will be estimated by the laboratory technicians by counting the number of asexual parasites per 200 leukocytes, assuming a leukocyte count of 8,000/μl. All patients who have a positive screening thick smear with a parasite density of > 2000/ul (> 50 parasites per 200 leucocytes) and < 200,000/ul (< 5000 parasites per 200 leucocytes) will be referred to the study clinic for further evaluation. If the patient satisfies the selection criteria, they will be enrolled in the study. All patients who do not satisfy the selection criteria and are excluded from study enrolment will be referred back to the outpatient department for appropriate care.

## 5.3 Selection criteria

On day 0, patients with symptoms suggestive of malaria and a positive screening thick blood smear will be assessed for the following selection criteria:

1. Not previously enrolled in this study
2. Age 1 – 10 years
3. Weight > 10 kg
4. Fever (> 37.5ºC axillary) or history of fever in the previous 24 hours
5. Absence of any history of serious side effects to study medications
6. No evidence of a concomitant febrile illness in addition to malaria
7. Provision of informed consent (appendix 5) and ability to participate in 28-day follow-up (patient has easy access to health unit)
8. No danger signs or evidence of severe malaria defined as:

- Unarousable coma (if after convulsion, > 30 min)
- Repeated convulsions (> 2 within 24 h)
- Recent convulsions (1-2 within 24 h)
- Altered consciousness (confusion, delirium, psychosis, coma)
- Lethargy
- Unable to drink or breast feed
- Vomiting everything
- Unable to stand/sit due to weakness
- Severe anaemia (Hb < 5.0 g/dL)
- Respiratory distress (laboured breathing at rest)
- Jaundice (yellow coloring of eyes)

Patients fulfilling these criteria will be assigned a study number and will be referred to the laboratory. A fingerprick blood sample will be obtained to prepare thick and thin blood smears, and for measurement of haemoglobin. After going to the laboratory, the subjects will be referred to the study nurse for treatment allocation and treatment with the study medications. Patients must also meet the following criteria:

1. Absence of repeated vomiting of study medications on day 0

Results of the Giemsa-stained thick and thin blood smears obtained on day 0 will not be available until after the patients have been treated and discharged from the clinic. Patients will return to the clinic on day 1 and will be excluded from the study if the following inclusion criteria are not met:

1. *P. falciparum* mono-infection
2. Parasite density > 2000/ul and < 200,000/ul

# 6.0 BASELINE EVALUATION AND TREATMENT ALLOCATION

## 6.1 Baseline evaluation and procedures

On day 0, patients fulfilling the selection criteria will be assigned a study number and will undergo a complete history and physical examination (Appendix 9). Patients will be referred to the laboratory to obtain a fingerprick blood sample for repeat thick blood smear, thin blood smear, haemoglobin measurement and to save of filter paper for future molecular testing. After going to the laboratory, the patients will be referred to the study nurse for treatment.

## 6.2 Treatment group assignment

Patients will be randomly assigned to one of the two treatment groups (AQ+AS or coartemether). Randomization will be done according to a pre-determined randomization list. Treatment allocation and administration of medications will be performed by the study nurse.

## 6.3 Treatment allocation

To allocate subjects to the appropriate treatment group, the study nurse will select the next available treatment number and corresponding study regimen. The study nurse will record the data and time of treatment assignment and the patient's study number.

## 6.4 Study intervention

**Table 3. Drug formulation and labelling**

| **Drug** | **Trade name (Manufacturer)** | **Class** |
| --- | --- | --- |
| Amodiaquine (200mg base) | Camoquin (Parke-Davis) | 4-aminoquinoline |
| Artesunate (50mg) | Arsumax (Sanofi) | artemisinin derivative |
| Artemether + lumefantrine  (20mg/120 mg) | Coartem (Novartis) | artemisinin derivative + dibutyl-aminoethanol |

### 6.4.1 Dosing of study medications

All subjects will receive two medications in the morning and one medication in the evening for 3 days. Subjects randomized to the AQ+AS group will receive AQ and AS in the morning and lactose placebo tablets in the evening. AQ will be given as 25 mg/kg over 3 days (10 mg/kg on day 0, 10 mg/kg on day 1, and 5 mg/kg on day 2) and AS will be given as 12 mg/kg over 3 days (4 mg/kg/day). AQ+AS subjects will also be given lactose placebo tablets given in the evening over 3 days, dosed similarly to weight-based guidelines for coartemether. Subjects randomized to the coartemether group will receive coartemether twice daily for 3 days, given in fixed dose tablets (20 mg artemether + 120 mg lumefantrine) according to weight-based guidelines. Coartemether subjects will also be given lactose placebo tablets in the morning for 3 days, dosed similarly to weight-based guidelines for AQ. Details of the dosing schedule are included in Appendix 6.

## 6.5 Treatment administration and blinding

Study medications will be administered according to weight-based guidelines (Appendix 6). Study medications will not be identical in appearance or taste, but the number of doses received will be similar (2 different medicines in the morning, 1 medicine in the evening) for patients in both treatment groups. Patients will not be informed of their treatment regimen, and all study staff involved in the assessment of patient outcomes, including the study clinicians (responsible for clinical assessment and measurement of temperature) and laboratory technicians (responsible for reading thick blood smears and determining parasite density) will be blinded to the treatment group assignments

Both the morning and evening doses of the study medication will be administered in the clinic. Study medications given to young children will be crushed, mixed with water, and administered as slurry. Study medications administered to older children and adults will be given as tablets or fractions of tablets to be taken orally with a glass of water. The study nurse will directly observe consumption of study medications. Patients will be observed for 30 minutes to ensure that the medications are not vomited. Any patient who vomits the medication within 30 minutes of administration will be retreated with a second dose. Any patient who vomits repeatedly (> 3 times) will be treated with/referred for treatment with parenteral quinine and recoded as having complicated malaria.

On day 0, the first dose of study medication will be administered before 11.00 a.m. and the second dose will be administered in the evening, approximately 8 hours later. On days 1 and 2, the first dose of study medication will be administered before 9.00 a.m. and the second dose will be administered in the evening, approximately 12 hours later.

Study participants will be free to either wait at the clinic or go home and return in the evening for the evening dose of study medications. Participants who opt to wait at the clinic shall wait in a specially designated waiting area. Participants will be reimbursed for transport to and from the clinic and for lunch (those who opt to wait at the clinic for the evening dose of medications). Home visitors shall actively follow patients who do not come for scheduled appointments.

## 6.6 Additional medications

On day 0, patients will receive paracetamol (10mg/kg) to take every 8 hours until the resolution of fever. Patients found to have uncomplicated malaria and a concomitant illness will be treated for both and followed up according to the study protocol. For patients with anaemia (Hb < 10 g/dL), we will follow Integrated Management of Childhood Illness (IMCI) guidelines: anaemic children will be treated with iron sulphate (100 mg po qD for 2 weeks) and mebendazole (only children > 1 year of age; 250 mg age 1-2 years; 500 mg > 2 years age; treated no more frequently than every 6 months).

# 7.0 FOLLOW-UP EVALUATION AND PROCEDURES

## 7.1 Follow-up schedule

Patients will be asked to return to the clinic for follow-up on days 1, 2, 3, 7, 14, 21, 28, and any unscheduled day that they feel ill. All patients will be reimbursed the cost of their transport to and from the clinic. At enrolment, details about the location of the patient’s residence will be obtained and if a subject does not return for a scheduled clinic follow-up appointment, the study personnel shall visit them at home. Patients who return on day 1 and fail to fulfil the criteria of *P. falciparum* mono-infection with a parasite density of > 2000 parasites/ul and < 200,000 parasites/ul will be excluded from further study and referred to OPD for management. At each repeat visit, temperature will be measured and a focused physical examination will be performed. A finger prick blood sample will be obtained on days 2, 3, 7, 14, 21 and 28 (and any extra day) to repeat thick blood smears and to save on filter paper. Haemoglobin will be re-evaluated on day 28 or at the time of clinical treatment failure.

# Table 4. Follow-up Schedule

|  | **Day 0** | **Day 1** | **Day 2** | **Day 3** | **Day 7** | **Day 14** | **Day 21** | **Day 28** | **Extra Day** |
| --- | --- | --- | --- | --- | --- | --- | --- | --- | --- |
| **Study medications** | X | X | X |  |  |  |  |  |  |
| **History** | X | X | X | X | X | X | X | X | X |
| **Temperature measurement** | X | X | X | X | X | X | X | X | X |
| **Physical exam** | X | X | X | X | X | X | X | X | X |
| **Thick blood smear** | X |  | X | X | X | X | X | X | X |
| **Thin blood smear** | X |  |  |  |  |  |  |  |  |
| **Filter paper sample** | X |  | X | X | X | X | X | X | X |
| **Haemoglobin** | X | † | † | † | † | † | † | X | † |
| **Assessment for adverse drug event** | X | X | X | X | X | X | X | X | X |

X = perform this task

† Performed on day of clinical failure

## 7.2 Classification of treatment outcome and management of treatment failure

Patients will be followed for 28 days and will have treatment outcomes assessed according to the WHO 2003 guidelines (Appendix 7). Patients who require clinical rescue therapy (ETF, LCF, or LPF) will be treated with quinine 10 mg/kg orally three times a day for 7 days. Any patient who is diagnosed with severe malaria or danger signs during follow-up, will be referred for appropriate treatment with parenteral quinine at the local facility or hospital. Patients not admitted to the hospital will receive oral quinine to complete a 7-day course of therapy. If a patient requires clinical rescue therapy, formal study follow-up will end, and the patient will be followed up at the discretion of the study physician. Haemoglobin measurement will be repeated on the day the patient receives clinical rescue therapy.

## 7.3 Exclusion after enrollment and loss to follow-up

Patients will be excluded from the study and not have their treatment outcome assessed if any of the following occurs after enrollment:

- 1. Use of antimalarial drugs outside of the study protocol.
  2. Development of a febrile illness (e.g. pneumonia, dysentery, measles) concomitantly with parasitaemia which interferes with outcome classification.
  3. Withdrawal of informed consent.
  4. Loss to follow-up: Patients who fail to attend a follow-up visit and are unable to be located within 24 hours on Days 1-3 or within 48 hours on Days 4-28.

## 7.4 Management of non-malarial illnesses

Patients who are found to have illnesses other than malaria during standard 28-day follow-up will receive standard-of-care treatment in the clinic, according to standardized algorithms, or will be referred to OPD for the appropriate care. Routine use of non-study medications with antimalarial activity, including tetracycline, antifolate, and macrolide antibiotics, will be avoided when acceptable alternatives are available.

# 8.0 ASSESSMENT FOR ADVERSE EVENTS

## 8.1 Definitions

An adverse event is defined as "any untoward medical occurrence in a patient or clinical investigation subject administered a pharmaceutical product that does not necessarily have a causal relationship with this treatment" (ICH Guidelines E2A). An adverse event can further be broadly defined as any untoward deviation from baseline health which includes:

- Worsening of conditions present at the onset of the study
- Deterioration due to the primary disease
- Intercurrent illness
- Events related or possibly related to concomitant medications

(International Centers for Tropical Disease Research Network Investigator Manual, Monitoring and Reporting Adverse Events, 2003).

A serious adverse event is defined as an experience that results in any of the following outcomes:

- Death during the period of study follow-up
- Life-threatening experience (one that puts a patient at immediate risk of death at the time of the event)
- Inpatient hospitalization during the period of study follow-up
- Persistent or significant disability or incapacity
- Specific medical or surgical intervention to prevent one of the other serious outcomes listed in the definition.

## 8.2 Identification of adverse events

At each follow-up visit (days 1, 2, 3, 7, 14, 21, 28, and any unscheduled day), study clinicians will assess patients according to a standardized clinical record form (appendix 12). A severity grading scale, based on toxicity grading scales developed by the WHO and the National Institutes of Health, Division of Microbiology and Infectious Diseases, will be used to grade severity of all symptoms, physical exam findings, and haemoglobin results (Appendices 9). Any new event, or an event present at baseline that is increasing in severity, will be considered an adverse event.

## 8.3 Reporting of adverse events

For each possible adverse event identified and graded as moderate, severe or life threatening, an adverse event report form will be completed (Appendix 12). An adverse event report form will not be completed for events classified as mild as these symptoms are common and difficult to distinguish from signs and symptoms due to malaria. The following information will be recorded for all adverse experiences that are reported:

1. Description of event
2. Date of event onset
3. Date event reported
4. Maximum severity of the event
5. Maximum suspected relationship of the event to study medication
6. Is the event serious?
7. Initials of the person reporting the event
8. Was the event episodic or intermittent in nature?
9. Outcome
10. Date event resolved

## 8.4 Reporting of serious adverse events

Guidelines for reporting of serious adverse events provided by the UCSF Committee for Human Research and the Ugandan National Council for Science and Technology will be followed.

# 9.0 STATISTICAL CONSIDERATIONS

## 9.1 Sample size calculations

The sample size calculations will be made for the study based on the estimated efficacy of the combination regimens to be studied in the given regions. For the purposes of sample size calculation, the risk of clinical rescue therapy will be the primary outcome. If the null hypothesis states that there is no difference in clinical outcome between the treatment groups, we plan to test the alternative hypothesis that treatment with an alternative combination regimen will decrease the likelihood of clinical failure at 28 days of follow-up. The risk of clinical rescue therapy with AQ+AS is estimated to be 40-50% based on previous data (UMSP, unpublished data). We plan to enroll 200 patients in each treatment arm at all of the study sites (400 patients per site, 1600 patients total). Based on an α = 0.05 (two-sided) and a power of 80%, allowing for 10% loss to follow-up, we will have the ability to detect a risk difference of approximately 15% between the two treatment groups.

## 9.2 Analytical plan

Data analysis will be primarily performed by the project epidemiologist using SPSS and STATA statistical software packages, with additional assistance from collaborators in San Francisco and the University of California, Berkeley, Department of Epidemiology.

Descriptive statistics will be used to summarise baseline characteristics of study patients. Because age is a strong effect modifier in malaria, an age-stratified subgroup analysis for efficacy outcomes is planned. Study subjects will be stratified by age (< 5 years and > 5 years) for analysis of treatment outcomes. Results for the two age groups will be presented independently. Data on efficacy data will be evaluated using a per-protocol analysis and will only included patients with treatment outcomes. Categorical variables will be compared between the treatment groups using chi-square tests or Fisher’s exact tests and continuous variables will be compared using t-tests. A p-value of < 0.05 will be considered significant. The risk of clinical and parasitological treatment failure after adjustment by genotyping shall be estimated using Kaplan-Meier survival analysis technique in accordance with the 2003 WHO protocol [26]. Data will be censored for new infections.

# 10.0 DATA COLLECTION AND MANAGEMENT

## 10.1 Data management

All clinical data will be recorded onto standardised case record forms by study clinicians. Laboratory data will be recorded in a laboratory record book by the study laboratory technicians and then transferred to the case record forms by the study clinicians. Data will be transferred from the case record forms into a computerised database (EPI INFO 6.04) by data entry personnel and will be double entered to verify accuracy of entry. Two back-up files of the database will be stored on compact discs after each data entry session. For quality control, check programs will be written into the database to limit the entry of incorrect data and ensure entry of data into required fields.

## 10.2 Data quality assurance and monitoring

All members of the study team will be educated in the study protocol prior to the onset of the trial. The study clinicians will complete case record forms at each patient visit. These forms will be reviewed by the study co-ordinator and site supervisors from the core facility for completeness and accuracy. For quality control of thick blood smear slide readings, expert microscopists who will be blinded to the patient’s treatment group will review a random sample of approximately 10% of slides. Study group meetings will be conducted by the co-ordinator once a week to assess progress of the study, address any difficulties, and provide performance feedback to the members of the study group. In addition members from the core facility will make regular visits to active study sites as needed.

## 10.3 Records

Case record forms will be provided for each subject. Participants will be identified by their initials and study identification number on the case record form. Patient names will not be entered into the computerised database. All patient record forms will be kept in individual files in a secure filing cabinet in the study clinic. All corrections will be made on case record forms by striking through the incorrect entry with a single line and entering the correct information adjacent to it. The correction will be initialled and dated by the investigator. Any requested information that is not obtained as specified in the protocol will have an explanation noted on the case record form as to why the required information was not obtained. Additional records will be kept in the clinical and laboratory record books at the core facility in Kampala. The investigators will allow all requested monitoring visits, audits or reviews.

# 11.0 LABORATORY PROCEDURES

## 11.1 Blood smears

Thick and thin blood smears will be stained with 2% Giemsa. Thick blood smears will be evaluated for the presence of parasitemia, including asexual forms and gametocytes. Parasite densities will be calculated from thick blood smears by counting the number of asexual parasites per 200 leukocytes (or per 500, if the count is <10 parasites/200 leukocytes), assuming a leukocyte count of 8,000/μl. A thick blood smear will be considered negative when the examination of 100 high power fields does not reveal asexual parasites. Thin blood smears will be evaluated to determine parasite species.

## 11.2 Haemoglobin measurement

Haemoglobin will be measured from fingerprick blood samples using a portable spectrophotometer (HemoCue, Anglom, Sweden).

## 11.3 Molecular studies

Blood samples will be collected from patients on days 0, 2, 3, 7, 14, 21, 28 and on any unscheduled day that the patient presents with clinical deterioration or recurrent fever. Blood will be placed onto filter paper in approximately 25 ul aliquots per blood spot (4 blood spots per sample). The samples will be labelled, air-dried and stored in small, sealed sample bags at ambient temperature. Parasite DNA will subsequently be removed from the filter paper and prepared for molecular analysis using a chelex extraction method. Genotyping of parasites collected at baseline (day 0) and during follow-up will be done to distinguish between true recrudescence and reinfection with new parasites. Briefly, the block 3 region of the merozoite surface protein-2 gene will be amplified using nested PCR and characterized based on sequence and size polymorphisms identified by restriction endonuclease digestion and gel electrophoresis. Genotyping patterns on the day of repeat therapy will be compared with those at treatment initiation using GelCompar II software (Applied Maths). This laboratory work will be conducted in the UCSF laboratory directed by Dr. Phil Rosenthal and in the Makerere University laboratory directed by Dr. Fred Kironde.

# 12.0 PROTECTION OF HUMAN SUBJECTS

## 12.1 Institutional Review Board (IRB) review and informed consent

This protocol and the informed consent documents, including any additional educational or recruitment material, will be reviewed and approved by the institutional review board of the Ugandan National Council of Science and Technology and the UCSF Committee for Human Research before the trial begins. Any amendments or modifications to this material will also be reviewed and approved by the IRBs prior to implementation.

## 12.2 Risks and discomforts

### 12.2.1 Privacy

Care will be taken to protect the privacy of subjects, as described in this protocol. However, there is a risk that others may inadvertently see patients’ medical information, and thus their privacy compromised.

### 12.2.2 Risks of randomization

This will be a randomized trial, and some treatment arms may prove to be more or less efficacious, more or less well tolerated, and/or more or less safe than others. Thus, there is the risk that patients will be randomized to less efficacious, less well tolerated, and/or less safe treatment regimens.

### 12.2.3 Fingerprick blood draws

Risks include pain, transient bleeding and soft-tissue infection.

### 12.2.4 Risk of amodiaquine

AQ has been described as “very well tolerated” for routine use, [27] and it was widely used for chemoprophylaxis against malaria in the past. However, prophylactic use was discontinued due to rare instances of agranulocytosis, aplastic anaemia, and hepatotoxicity, principally associated with use for malarial chemoprophylaxis in travellers. [7, 27] Reported rates of serious reactions to AQ in the UK were 1:2100 blood dyscrasias, 1:31,000 deaths from blood dyscrasias, and 1:15,650 serious hepatotoxicity. [28] Toxicities with short-term use for treatment are expected to be much lower, although data are limited. [7-11] In a review of 40 published and unpublished clinical trials, no severe or life-threatening adverse event was noted.[7] Considering tolerability in 488 AQ-treated patients, gastrointestinal toxicities and pruritis were most commonly reported, and the incidence of adverse events was similar among patients treated with AQ, CQ, and SP.[7] In Uganda, no serious toxicities were observed with AQ monotherapy (131 treatments). [11]

### 12.2.5 Risk of artemisinins

Artemisinin derivatives have now been extensively studied, and they are remarkable for a lack of serious toxicity when used for the treatment of malaria [13]. Considering all artemisinins, 15% (12,463) of the patients enrolled in all published antimalarial drug trials over the past 50 years have received an artemisinin compound, and there are more trials on these compounds than on any other antimalarials (N. White, unpublished communication). In addition to formal studies, artemisinins have now been widely used, with well over a million treatments, mostly of AS, in Southeast Asia. The only serious toxicity which has emerged in detailed prospective clinical evaluations is a low risk of type 1 hypersensitivity reactions (estimated risk 1:2833, 95% CI 1:1362-1:6944) [29]. Electrocardiograms and detailed neurological, audiometric, and neurophysiological tests have failed to show any evidence for cardiac or neurological toxicity in humans (see below for more details) [30-33]. Considering oral AS, adverse events appear to be rare. The package insert for AS (Sanofi-Winthrop, France) notes only two potential laboratory abnormalities “in a few cases”, lowering of reticulocyte count and slight increases in transaminases.

Animal studies have led to some concerns over artemisinins, particularly regarding cardiac and neurological effects, and reproductive toxicity. As slight QT prolongation was observed in dogs treated with high doses, detailed electrocardiographic studies have been conducted in humans during treatment for falciparum malaria [34]; [31-33] [35]. Taking into account effects of malaria, no significant effects of artemisinins on the QT interval were identified.

The neurological effects of artemisinins have been very extensively studied. In mice, rats, dogs, and monkeys, high dosages of intramuscular artemether and arteether produce an unusual and selective pattern of damage to certain brainstem nuclei, particularly those of the auditory and vestibular systems [36-47]. AS is transformed in vivo to dihydroartemisinin, which is the most neurotoxic of the artemisinin derivatives [48-50]. However, in the animal models, orally administered AS and dihydroartemisinin are considerably less neurotoxic than intramuscular artemether or arteether. Differences in toxicity are explained by differences in pharmacokinetics of different compounds and different routes of administration [40, 44, 46, 51]. Neurotoxicity results from the long-lasting blood concentrations that follow intramuscular injection of the oil-soluble compounds, artemether and arteether. Oral administration of artemether or arteether, which provides much more rapid absorption and elimination than intramuscular dosing, leads to markedly less neurotoxicity in mice, although oral artemether can be made more neurotoxic by giving the drug in small repeated doses to simulate the constant exposure that follows intramuscular injection [44]. Artesunate is much less toxic than arteether in rats when administered intramuscularly [45] or orally [41, 46, 52]. Importantly, with high dose intramuscular injections of artemether and arteether, clinical assessment of mice was a sensitive indicator of neurotoxicity; no mice with normal clinical exams showed histopathology [50].

In animals, there is clear evidence of death of embryos and some evidence for morphological abnormalities when artemisinin derivatives are administered in early pregnancy.[15] In addition, when artemisinins are given later in pregnancy, there is some evidence of adverse effects on fetal body weight and survival. Data on administration of artemisinin compounds during the second or third trimesters (607 pregnancies) do not suggest evidence of treatment-related adverse pregnancy outcomes, and similar data in 124 first trimester pregnancies indicated normal outcomes. However, few pregnancies have been evaluated, and further investigation on the reproductive safety of artemisinins is needed.

The artemisinin derivatives are remarkably well tolerated in humans. In a clinical safety review of 108 studies including 9,241 patients, no serious adverse events or significant toxicity was reported [14]. In addition, a systematic review of artemisinin derivatives for treating uncomplicated malaria, including 41 studies of 5,240 patients, showed no evidence of harmful effects related to artemisinin derivatives [53]. Clinical studies have shown no convincing evidence for neurotoxicity after treatment with artemisinin derivatives, though neurological effects of acute malaria are common. One letter described ataxia and slurred speech after AS therapy, but these findings were consistent with the course of severe malaria [54]. To specifically evaluate for potential artemisinin-associated auditory toxicity in humans, van Vugt et al. performed clinical neurological evaluations, audiometry and early latency auditory evoked responses in 79 patients treated with multiple doses of artemether or artesunate and 79 matched controls in Thailand, and no evidence of auditory toxicity was detected [32]. Comparisons of patients who had received multiple courses of artemisinin derivatives with age-matched untreated controls showed no significant differences in clinical, audiometric, or auditory evoked potential measurements [30, 32]*.* Even considering the most worrisome dosing regimen, there is no evidence that clinical use of intramuscular artemether has caused neurotoxicity. In a new report, four independent neuropathologists examined the brains of patients who died after treatment with intramuscular artemether, and there was no evidence for the characteristic pattern of neuropathological change seen in the animal studies [55]. These results suggest a wide margin of safety for artemisinins in clinical use, particularly when given orally, particularly for water soluble compounds, and most particularly for the most widely studied water-soluble agent, AS.

### 12.2.6 Artemether-Lumefantrine (Coartemether)

Coartemether (Coartem and Riamet; Novartis) has been extensively studied through GCP standardized preclinical and clinical trials and was added to the WHO Essential Medicines List in 2002 [56]. It has been approved for use against malaria in both developing and developed (e.g. Switzerland) countries. The drug appears to be very well tolerated, especially in comparison to other antimalarials and antimalarial combinations including chloroquine, quinine, and mefloquine+artesunate. A clinical safety review of children under 12 years of age showed that the most common adverse events were abdominal pain, cough, anorexia, headache, vomiting, and diarrhea (all seen in 5-12% of subjects). [57]

An integrated review of toxicity in 1869 patients (611 under age 13) showed the most commonly reported adverse events were gastrointestinal disturbances (abdominal pain, anorexia, nausea, vomiting diarrhea), headache, and dizziness. Rash and pruritis were reported in <2% of patients. No serious or persistent neurological toxicities were linked to coartemether therapy. Of 20 severe adverse events in 1869 patients, 19 were likely attributable to underlying malaria or concomitant illness, and one was possibly related to coartemether use (hemolytic anaemia in a 35-year-old 13 days after the last administered dose) [58]. One concern addressed in studies of coartemether was possible cardiac arrhythmogenic potential based on similarities in the chemical structures of lumefantrine and halofantrine. Halofantrine can cause defects in cardiac conduction, particularly a marked QT prolongation that can produce arrhythmias. In 713 patients treated with lumefantrine and followed with serial electrocardiograms, no adverse clinical cardiac events were recorded. Although trials have been limited to date, no serious cardiotoxicity or neurotoxicity has been reported with the use of coartemether [20, 22, 32, 59].

### 12.2.7 Risk of Quinine

Quinine is the standard drug for the treatment of severe malaria throughout Africa and is also the standard drug for the treatment of falciparum malaria in the U.S. It will be the treatment for patients who fail therapy with any study regimen. Quinine can commonly cause tinnitus, headache, nausea, dizziness, flushing, and visual disturbances. These symptoms, termed “cinchonism”, do not warrant discontinuing therapy unless they are severe. Less common toxicities include vomiting, diarrhea, and abdominal pain. Rare toxicities include skin rashes, urticaria, angioedema, bronchospasm, hemotologic abnormalities (hemolysis, leukopenia, agranulocytosis, and thrombocytopenia). Quinine can cause hypoglycemia, especially in pregnancy. Cardiovascular toxicity is seen principally with intravenous quinine, which will not be used in this study.

## 12.3 Compensation

The patients/patients’ families will receive reimbursement for transportation costs to and from the clinic. In addition, all clinic visits, antimalarial medication, and the evaluation and treatment for some routine medical problems encountered during follow-up will be provided free of charge. Medical care that the patient receives which is unrelated to malaria will remain the primary responsibility of the patient, parent or guardian, although routine medical problems will generally be managed by the study at no cost to the patient.

## 12.4 Consent procedures

All screening interviews will be conducted in the native language of the patients by the study personnel (with a translator if necessary). Consent forms will be provided to the patients / parents or guardians for their review. The study clinicians at the clinic will seek formal consent. The patients / parents or guardians will be asked to sign consent to participate in a research study. The informed consent will describe the purpose of the study, the procedures to be followed, and the risks and benefits of participation. If a patient, parent or guardian is unable to read or write, his/her fingerprint will be used in substitute for a signature, and a signature from a witness to the informed consent discussion will be obtained. Patients / parents or guardians will be informed that participation in the study is completely voluntary and that they may withdraw from the study at any time.

## 12.5 Alternatives

Individuals whose parents or guardians choose not to participate in this study will not be enrolled. They will receive standard care for medical problems as they arise at the government health dispensaries or other medical facilities in the UMSP sentinel sites.

## 12.6 Confidentiality of records

Patients, parents and guardians will be informed that participation in a research study may involve a loss of privacy. All records will be kept as confidential as possible. Patients will be identified primarily by their study number and patient names will not be entered into the computerized database. No individual identities will be used in any reports or publications resulting from the study.

# 13.0 TIMETABLE

**Table 6. Timetable for Study Activities**

|  | **2004** | | | | | | | | | | | |
| --- | --- | --- | --- | --- | --- | --- | --- | --- | --- | --- | --- | --- |
|  | **J** | **F** | **M** | **A** | **M** | **J** | **J** | **A** | **S** | **O** | **N** | **D** |
| **Finalization/approval of study protocol** |  |  |  |  |  | X | X |  |  |  |  |  |
| **Database design** |  |  |  |  |  |  | X |  |  |  |  |  |
| **Personnel training** |  |  |  |  |  |  | X |  |  |  |  |  |
| **Pilot study** |  |  |  |  |  |  | X |  |  |  |  |  |
| **Recruitment** |  |  |  |  |  |  | X | X | X | X | X |  |
| **Follow-up** |  |  |  |  |  |  | X | X | X | X | X |  |
| **Data entry/analysis** |  |  |  |  |  |  | X | X | X | X | X |  |
| **Manuscript preparation** |  |  |  |  |  |  |  |  |  |  | X | X |

# 14.0 REFERENCES

*1. Campbell, C.C., Challenges facing antimalarial therapy in Africa. Journal of Infectious Diseases, 1991.* ***16****: p. 1207-1211.*

*2. Trape, J.F., The public health impact of chloroquine resistance in Africa. Am J Trop Med Hyg, 2001.* ***64****(1,2 Suppl): p. 12-17.*

*3. Staedke, S.G., et al., Combination therapies for uncomplicated falciparum malaria in Kampala, Uganda: a randomized clinical trial. submitted for publication, 2004.*

*4. Brockman, A., et al., Plasmodium falciparum antimalarial drug susceptibility on the north-western border of Thailand during five years of extensive use of artesunate-mefloquine. Trans R Soc Trop Med Hyg, 2000.* ***94****(5): p. 537-44.*

*5. Price, R.N., et al., Artesunate/mefloquine treatment of multi-drug resistant falciparum malaria. Trans R Soc Trop Med Hyg, 1997.* ***91****(5): p. 574-7.*

*6. Attaran, A., et al., WHO, the Global Fund, and medical malpractice in malaria treatment. Lancet, 2004.* ***363****(9404): p. 237-40.*

*7. Olliaro, P., et al., Systematic review of amodiaquine treatment in uncomplicated malaria. Lancet, 1996.* ***348****: p. 1196-1201.*

*8. van Dillen, J., et al., A comparison of amodiaquine and sulfadoxine-pyrimethamine as first-line treatment of falciparum malaria in Kenya. Trans R Soc Trop Med Hyg, 1999.* ***93****(2): p. 185-8.*

*9. Brasseur, P., et al., Amodiaquine remains effective for treating uncomplicated malaria in west and central Africa. Trans R Soc Trop Med Hyg, 1999.* ***93****(6): p. 645-50.*

*10. Gorissen, E., et al., In vivo efficacy study of amodiaquine and sulfadoxine/ pyrimethamine in Kibwezi, Kenya and Kigoma, Tanzania. Trop Med Int Health, 2000.* ***5****(6): p. 459-63.*

*11. Staedke, S.G., et al., Amodiaquine, sulfadoxine/pyrimethamine, and combination therapy for treatment of uncomplicated falciparum malaria in Kampala, Uganda: a randomised trial. Lancet, 2001.* ***358****(9279): p. 368-74.*

*12. Dorsey, G., et al., Predictors of chloroquine treatment failure in children and adults with falciparum malaria in Kampala, Uganda. Am J Trop Med Hyg, 2000.* ***62****(6): p. 686-92.*

*13. Meshnick, S.R., T.E. Taylor, and S. Kamchonwongpaisan, Artemisinin and the antimalarial endoperoxides: from herbal remedy to targeted chemotherapy. Microbiol Rev, 1996.* ***60****(2): p. 301-15.*

*14. Ribeiro, I.R. and P. Olliaro, Safety of artemisinin and its derivatives. A review of published and unpublished clinical trials. Med Trop (Mars), 1998.* ***58****(3 Suppl): p. 50-3.*

*15. World Health Organization, Assessment of the safety of artemisinin compounds in pregnancy. 2003.*

*16. Adjuik, M., et al., Amodiaquine-artesunate versus amodiaquine for uncomplicated Plasmodium falciparum malaria in African children: a randomised, multicentre trial. Lancet, 2002.* ***359****(9315): p. 1365-72.*

*17. AFRO, Global AMDP database. (http://rbm.who.int/amdp/amdp_afro.htm).*

*18. de Vries, P.J. and T.K. Dien, Clinical pharmacology and therapeutic potential of artemisinin and its derivatives in the treatment of malaria. Drugs, 1996.* ***52****(6): p. 818-36.*

*19. Bunnag, D., et al., Clinical trial of artesunate and artemether on multidrug resistant falciparum malaria in Thailand. A preliminary report. Southeast Asian J Trop Med Public Health, 1991.* ***22****(3): p. 380-5.*

*20. Bindschedler, M., et al., Comparison of the cardiac effects of the antimalarials co-artemether and halofantrine in healthy participants. Am J Trop Med Hyg, 2002.* ***66****(3): p. 293-8.*

*21. Lefevre, G., et al., A clinical and pharmacokinetic trial of six doses of artemether-lumefantrine for multidrug-resistant Plasmodium falciparum malaria in Thailand. Am J Trop Med Hyg, 2001.* ***64****(5-6): p. 247-56.*

*22. Omari, A.A., C. Gamble, and P. Garner, Artemether-lumefantrine for uncomplicated malaria: a systematic review. Trop Med Int Health, 2004.* ***9****(2): p. 192-9.*

*23. World Health Organization, Global Anti-malarial drug policy database-AFRO. 2003.*

*24. Snow, R.W., E. Eckert, and A. Teklehaimanot, Estimating the needs for artesunate-based combination therapy for malaria case-management in Africa. Trends Parasitol, 2003.* ***19****(8): p. 363-9.*

*25. World Health Organization, Assessment of therapeutic efficacy of antimalarial drugs for uncomplicated falciparum malaria in areas with intense transmission. Technical Report Series, ed. W.D.o.C.o.T. Diseases. 1996, Geneva.*

*26. World Health Organization, Assessment and monitoring of antimalarial drug efficacy for the treatment of uncomplicated falciparum malaria. 2003. p. http://www.emro.who.int/rbm/publications/protocolwho.pdf.*

*27. Luzzi, G.A. and T.E.A. Peto, Adverse effects of antimalarials. Drug Safety, 1993.* ***8****(4): p. 295-311.*

*28. Phillips-Howard, P.A. and L.J. West, Serious adverse drug reactions to pyrimethamine-sulphadoxine, pyrimethamine-dapsone and to amodiaquine in Britain. J R Soc Med, 1990.* ***83****(2): p. 82-5.*

*29. Leonardi, E., et al., Severe allergic reactions to oral artesunate: a report of two cases. Trans R Soc Trop Med Hyg, 2001.* ***95****(2): p. 182-3.*

*30. Kissinger, E., et al., Clinical and neurophysiological study of the effects of multiple doses of artemisinin on brain-stem function in Vietnamese patients. Am J Trop Med Hyg, 2000.* ***63****(1-2): p. 48-55.*

*31. Tran, T.H., et al., A controlled trial of artemether or quinine in Vietnamese adults with severe falciparum malaria. N Engl J Med, 1996.* ***335****(2): p. 76-83.*

*32. van Vugt, M., et al., A case-control auditory evaluation of patients treated with artemisinin derivatives for multidrug-resistant Plasmodium falciparum malaria. Am J Trop Med Hyg, 2000.* ***62****(1): p. 65-9.*

*33. Price, R., et al., Adverse effects in patients with acute falciparum malaria treated with artemisinin derivatives. Am J Trop Med Hyg, 1999.* ***60****(4): p. 547-55.*

*34. van Vugt, M., et al., No evidence of cardiotoxicity during antimalarial treatment with artemether-lumefantrine. Am J Trop Med Hyg, 1999.* ***61****(6): p. 964-7.*

*35. Touze, J.E., et al., The effects of antimalarial drugs on ventricular repolarization. Am J Trop Med Hyg, 2002.* ***67****(1): p. 54-60.*

*36. Nosten, F., et al., Cardiac effects of antimalarial treatment with halofantrine. Lancet, 1993.* ***341****(8852): p. 1054-6.*

*37. Brewer, T.G., et al., Neurotoxicity in animals due to arteether and artemether. Trans R Soc Trop Med Hyg, 1994.* ***88 Suppl 1****: p. S33-6.*

*38. Brewer, T.G., et al., Fatal neurotoxicity of arteether and artemether. Am J Trop Med Hyg, 1994.* ***51****(3): p. 251-9.*

*39. Kamchonwongpaisan, S., et al., Artemisinin neurotoxicity: neuropathology in rats and mechanistic studies in vitro. Am J Trop Med Hyg, 1997.* ***56****(1): p. 7-12.*

*40. Petras, J.M., et al., Arteether: risks of two-week administration in Macaca mulatta. Am J Trop Med Hyg, 1997.* ***56****(4): p. 390-6.*

*41. Nontprasert, A., et al., Assessment of the neurotoxicity of parenteral artemisinin derivatives in mice. Am J Trop Med Hyg, 1998.* ***59****(4): p. 519-22.*

*42. Genovese, R.F., et al., Dose-dependent brainstem neuropathology following repeated arteether administration in rats. Brain Res Bull, 1998.* ***45****(2): p. 199-202.*

*43. Classen, W., et al., Differential effects of orally versus parenterally administered qinghaosu derivative artemether in dogs. Exp Toxicol Pathol, 1999.* ***51****(6): p. 507-16.*

*44. Li, Q.G., et al., Arteether toxicokinetics and pharmacokinetics in rats after 25 mg/kg/day single and multiple doses. Eur J Drug Metab Pharmacokinet, 1999.* ***24****(3): p. 213-23.*

*45. Genovese, R.F., D.B. Newman, and T.G. Brewer, Behavioral and neural toxicity of the artemisinin antimalarial, arteether, but not artesunate and artelinate, in rats. Pharmacol Biochem Behav, 2000.* ***67****(1): p. 37-44.*

*46. Nontprasert, A., et al., Studies of the neurotoxicity of oral artemisinin derivatives in mice. Am J Trop Med Hyg, 2000.* ***62****(3): p. 409-12.*

*47. Smith, S.L., et al., The role of glutathione in the neurotoxicity of artemisinin derivatives in vitro. Biochem Pharmacol, 2001.* ***61****(4): p. 409-16.*

*48. Wesche, D.L., et al., Neurotoxicity of artemisinin analogs in vitro. Antimicrob Agents Chemother, 1994.* ***38****(8): p. 1813-9.*

*49. Newton, P., et al., Antimalarial bioavailability and disposition of artesunate in acute falciparum malaria. Antimicrob Agents Chemother, 2000.* ***44****(4): p. 972-7.*

*50. Nontprasert, A., et al., Neuropathologic toxicity of artemisinin derivatives in a mouse model. Am J Trop Med Hyg, 2002.* ***67****(4): p. 423-9.*

*51. Genovese, R.F., et al., Acute high dose arteether toxicity in rats. Neurotoxicology, 1999.* ***20****(5): p. 851-9.*

*52. Nontprasert, A., et al., Assessment of the neurotoxicity of oral dihydroartemisinin in mice. Trans R Soc Trop Med Hyg, 2002.* ***96****(1): p. 99-101.*

*53. McIntosh, H.M. and P. Olliaro, Artemisinin derivatives for treating uncomplicated malaria. Cochrane Database Syst Rev, 2000(2): p. CD000256.*

*54. Miller, L.G. and C.B. Panosian, Ataxia and slurred speech after artesunate treatment for falciparum malaria. N Engl J Med, 1997.* ***336****(18): p. 1328.*

*55. Hien, T.T., et al., Neuropathological assessment of artemether-treated severe malaria. Lancet, 2003.* ***362****(9380): p. 295-6.*

*56. World Health Organization, Position of WHO's Roll Back Malaria Department on malaria treatment policy. 2003(http://www.emro.who.int/rbm/).*

*57. Novartis, Coartem monograph, 3rd ed., January 2004. 2004.*

*58. Bakshi, R., et al., An integrated assessment of the clinical safety of artemether-lumefantrine: a new oral fixed-dose combination antimalarial drug. Trans R Soc Trop Med Hyg, 2000.* ***94****(4): p. 419-24.*

*59. van Agtmael, M.A., T.A. Eggelte, and C.J. van Boxtel, Artemisinin drugs in the treatment of malaria: from medicinal herb to registered medication. 1999.* ***20****: p. 199-205.*

# APPENDIX 1. PARTICIPANT SELECTION AND ENROLLMENT

Patients attending OPD aged 1 – 10 years with history of recent fever

Send to laboratory

Screening thick blood smear

Negative smear or parasitaemia > 2000 < 200,000 parasites/ul

< 2,000 or > 200,000 /ul

Refer patient to clinician

Refer patient back to OPD

No information collected

Complete Screening Form

Excluded Passed Initial Screening

Complete Informed Consent Form

Excluded

Refer patient back to OPD

Save screening form only

Assign Study Number

#### Excluded

Complete Case Record Forms

Excluded

Refer to the Laboratory

Hb < 5.0

Schedule Follow up Visits

Refer to the Study Nurse

Assign Treatment Number

Thin and thick smear read overnight

Meet additional inclusion criteria on Day 1?

*P. falciparum* monoinfection

Parasite density > 2000 <200000/ul

**Yes**

**No**

**Excluded on Day 1**

**(Treat as appropriate)**

**Continue with scheduled follow-up outlined in appendix 4.**

# Appendix 2: Patient Screening Form

**STUDY SITE CODE:________ STUDY NUMBER: ___________________________**

UMSP SCREENING FORM

| **1. Names:** | **2. Date: *(dd/mm/yy)*** | **3.Weight (kg):** |
| --- | --- | --- |
| **4. Age*: _______years __________months.** | **5. Gender: M _______ F_______** | |

****Include months only if age < 5 years, if age is above 5 years write*** “X”

| **SCREENING selection criteria**  ***Patients who are 1-10 years of age and have a positive screening thick blood smear.*** | | |
| --- | --- | --- |
| **inclusion criteria** | **yes** | **NO** |
| 6. Fever (> 37.5°C) or history of fever in previous 24 hours |  |  |
| 7. Weight > 10 kg |  |  |
| 8. Ability to participate in 28 day follow up. |  |  |
| **EXCLUSION CRITERIA** | **NO** | **YES** |
| 9. Previously participated in this study? |  |  |
| 10. History of serious side effects to study medications  *If present, indicate drug / side effect:*   Amodiaquine:________________________   Artesunate:__________________________   Coartemether:________________________ |  |  |
| 11. Evidence of severe malaria / danger signs  *If “ YES” indicate criteria. If “NO”, leave blank.*  Unarousable coma *(if after convulsion, > 30 min)*   Repeated convulsions *(> 2 within 24 h)*   Recent convulsions *(1-2 within 24 h)*   Altered consciousness *(confusion, delirium, coma)*   Lethargy   Unable to drink or breast feed   Vomiting everything   Unable to stand/sit due to weakness   Severe anaemia *(Hb < 5.0 g/dL)*   Respiratory distress *(laboured breathing at rest)*   Jaundice *(yellow coloring of eyes)* |  |  |
| 12. Evidence of concomitant febrile illness  *If “YES”, indicate illness. If “NO”, leave blank.*   Pneumonia/RTI  Measles   Otitis Media  UTI   Gastroenteritis  Other:_________________ |  |  |
| **INCLUSION CRITERIA** | **YES** | **NO** |
| 13. Provision of informed consent. |  |  |
| 14. Absence of persistent vomiting of study medications on day 0 |  |  |
| ***Complete prior to day 1 clinic visit.*** | | |
| 15. *P. falciparum* mono-infection |  |  |
| 16. Parasite density > 2000/ul and < 200,000/ul  *If “NO” specify density*  < 2000/ul  > 200,000/ul |  |  |

***If any of the responses fall into the shaded area, exclude the patient from the study***

# APPENDIX 3. UMSP ENROLLMENT FORM.

| **enrollment form** | | |
| --- | --- | --- |
| **1. Study**  **Number:** | **2. Treatment**  **Number:** | **3. Start Date:**  ***(dd/mm/yy)*** |

| 4. Patients name: |
| --- |
| 5. Father’s/Husband’s (if married) name: |
| 6. Mother’s/Wife’s (if married) name: |
| 7. Primary caregiver / guardian’s name and relationship (N/A if mother or father): |
| 8. Sub county of residence. |
| 9. Home parish: |
| 10. LC1/village: |
| 11. Home address and localising features: |
| 12. a) Phone number: Yes ___No ___  If yes: b) Phone number (s) and the owner(s): |
| Patient Information |
| 13. Does the patient use malaria preventive measures: Yes ___No ___Unknown ____  If yes, which measures are used?    a) Spray: Yes ___No ___  b) Coils: Yes ___No ___  c) Chemoprophylaxis: Yes ___No ___ 🡪 If yes, which drug? ___________________________________  d) Bed net: Yes ___No ____ 🡪 If yes, is the net treated? Yes ___No ___  🡪 How often does the patient sleep under the net?  Always____ Often \ Sometimes____  e) Other protective measures ______________________________________________________________ |

# APPENDIX 4. Critical steps

**Day 1.**

Evaluate patient and complete Case Record Form. Administer study medications (DOT).

**Day 2.**

Evaluate patient and complete Case Record Form. Administer study medications (DOT)

Collect thick blood smear and filter paper sample

**Day 3.**

Evaluate patient and complete Case Record Form. Collect thick blood smear and filter paper sample

**Day 7, 14, 21**. Evaluate patient and complete Case Record Form. Collect thick blood smear and filter paper sample

**Any Unscheduled Day (Day 4-27).**

Evaluate patient and complete Case Record Form. Collect thick blood smear with filter paper sample.

D2 Parasitaemia > D 0 parasitaemia

**ETF**

**Treat with oral quinine**

**Severe disease or danger signs**

Do urgent thick smear, FP sample and Haemoglobin

If patient has

1. Temperature > 37.5^0^C with parasitaemia

2. Parasite count >25% Day 0 count.

**ETF**

**Treat with oral quinine**

**Day 28.**

Evaluate patient and complete Case Record Form. Collect thick blood smear and filter paper sample.

**Severe disease or danger signs**

Do urgent thick smear, FP sample and Haemoglobin

Negative smear

**Continue study**

**Rx at your discretion.**

Negative smear

**Continue study Rx at your discretion.**

Positive blood smear

**ETF**

**Give/ refer for IV quinine Rx.**

Positive blood smear

**LCF**

**Give/ refer for IV Quinine Rx.**

**ACPR** No parasitaemia on day 28 irrespective of fever history or temperature.

**LPF** Parasitaemia D 28 with temperature < 37.5^0^C without being ETF or LCF.

# Day 2 > Day 0

## ETF

Rx with quinine

Refer for care?

# Day 2 < Day 0

Assess for DDx

Cont. study Rx

Refer for care?

# Yes

## ETF

Rx with quinine

'

If patient has

1. Temp > 37.5°C with parasitaemia

2. History of fever in the last 24 hours with parasitaemia.

**Do Haemoglobin and thin blood smear**

LCF

Treat with oral quinine

Patients who are LPF’s should be treated with oral quinine.

# Appendix 5. Informed Consent

**Study number: __________________________**

RESEARCH PARTICIPANT INFORMED CONSENT FORM

**Protocol Title:** Comparison of amodiaquine + artesunate and artemether + lumefantrine for treatment of uncomplicated malaria in Uganda: evaluation of efficacy, tolerability, and safety

**Site of Research:** UMSP Sentinel Sites, Uganda

**Sponsor:** Centers for Disease Control

**Principal Investigator**: Fred Wabwire-Mangen, MBChB, DTMH, MPH, PhD

**Date:** 22 September 2004

**PURPOSE OF THE STUDY**

This research study is being done to learn more about the treatment of malaria. We would like to know what the best and safest treatment is for malaria in Uganda. To do this, we are carrying out a research study to compare different combinations of malaria drugs. About 400 participants will be involved in this study. The study is conducted by the Uganda Malaria Surveillance Project (UMSP), which is a collaboration between the Ugandan Ministry of Health and academic partners from Uganda and the USA.

**HOW THE STUDY IS DONE**

Your child or the child under your care in the case of a legal guardian (hereafter referred to as “your child”), will be treated for malaria with amodiaquine + artesunate (AQ+AS) or artemether + lumefantrine (coartemether). All of these drugs are registered for the treatment of malaria in Uganda, but it is unclear which combination is the safest and most effective. After the treatment, your child will be followed for 28 days to see if the malaria infection is cured. If your child does not get better after treatment, treatment with quinine will be given. The treatment that your child will receive will be determined by a process of randomization. Randomization means that your child will be put into a group by chance, similar to pulling a number out of a hat. The chance of being placed into each of the treatment groups is one in two. You will not be told which treatment your child has been assigned to receive. When the project is completed, you can ask which treatment group was assigned. You are being asked to allow your child, to participate in this study for up to 28 days or until such a time as you or the study doctors decide that your child should no longer participate in the study. The study may be discontinued by the sponsor at any time, and for any reason. The study doctors may withdraw your child from the study for the following reasons:

1. If your child receives malaria medicines not prescribed by the study doctors
2. If your child develops a febrile illness in addition to malaria which makes it difficult for the doctors to tell which problem is causing the fever
3. If you chose to withdraw your consent to participate in the study
4. If we are unable to locate your child within 24 hours on days 1-3, or within 48 hours on days 4-28 of the study follow-up period.

PROCEDURES

- 1. The study doctors will examine your child today.
  2. A blood sample will be collected. A small amount of blood will be taken by fingerprick to examine for malaria parasites, to measure the blood count, to store blood samples on filter paper for future laboratory tests that will not impact on the health care your child.
  3. If the diagnosis of malaria is confirmed, and your child is eligible for the study, treatment with amodiaquine (Camoquin) + artesunate (Arsumax) (AQ+AS), or artemether + lumefantrine (coartemether) will be given.
  4. You will be asked to return to the clinic at least 7 more times over the next 4 weeks so that the success of the treatment can be judged. At each of the follow-up visits, the study doctors will examine your child. He/she will assess your child’s health and determine possible side effects to the study medications. At five of these visits, a small amount of blood will be taken by finger-prick to examine for malaria parasites and to save on filter paper. The blood counts will be measured again on the last day.
  5. If your child misses an appointment, the home health visitor will visit you at your home to find out why you missed the appointment and bring you and your child to the clinic for assessment.
  6. If, at any time, the treatment given to your child does not seem to be working well, it will be changed to quinine.
  7. There will be someone at the study clinic every day from 8:00 am to 5:00 pm and at night. Your child can come to the clinic for evaluation anytime that your child is ill during the next 28 days.

**RISKS AND DISCOMFORTS**

**Risks involved with the study treatments**

1. Side effects with some of these drugs may be more common when two drugs are taken together. Your child will be monitored closely after receiving treatment for malaria with the study medications for any possible side effects of the drugs and will receive appropriate medical care for any problem that happens during the course of the study.
2. Major side effects: Severe problems that have been reported include the following:
   1. amodiaquine (Camoquin) - lowering of blood counts, bone marrow failure, inflammation of the liver, and death
   2. artesunate (Arsumax) - lowering of the blood counts and inflammation of the liver
   3. coartemether (Coartem) - no serious neurological effects or clinically relevant alterations in laboratory parameters reported.

However, serious health problems, including death, have rarely been reported following treatment with the study medications.

1. Minor side effects. The following side effects have been reported in association with the study medications-
   1. amodiaquine (Camoquin) - nausea, vomiting, diarrhea, lethargy (tiredness)
   2. artesunate (Arsumax) - headache, nausea, vomiting, abdominal pain, diarrhea, dizziness, tinnitus (ringing noise in ears), neutropenia (low blood count), abnormalities of liver tests.
   3. co-artemether (Coartem) - gastrointestinal disturbances (stomach upset), rash, and pruritis (itching).
2. Severe malaria: Your child may develop malaria that is severe even after receiving treatment with study medications. If your child shows any evidence of severe malaria (including persistent vomiting, low blood counts, convulsions, confusion, or coma) they will be treated with quinine and referred for possible admission to hospital.
3. Unknown Risks: The research treatments may have side effects that no one knows about yet. The researchers will let you know if they learn anything that might make you change your mind about your child’s participation in the study.

**Risks involved in study procedures**

1. Randomization: Your child will be assigned to a treatment group by chance. The treatment your child receives may prove to be less effective or to have more side effects than the other study treatments or than other available treatments. This will not be known until after the study is completed.
2. Blood draws: The risks of drawing blood from a blood vessel (usually from a vein in the arm or hand) or from a fingerprick include temporary discomfort from the needle stick, bruising, skin infection, and fainting. The amount of blood removed will be too small to affect your child’s health.
3. Confidentiality: Participation in research may involve a loss of privacy, but information about your child will be handled as confidentially as possible. Medical information related to malaria will be collected on your child, but only the people working on the study will see it. Anyone assigned to review this study will be granted direct access to your child's medical records, if necessary, for verification of the study procedures and data. Records will be kept as confidential as possible.

**BENEFITS**

1. The potential benefit to your child is that the treatment received may prove to be more effective than the other study treatments or than other available treatments, although this cannot be guaranteed.
2. Your child will receive clinical care from the medical officers and nurses of the project staff in the study clinic. This will include care for unscheduled sick visits.
3. The knowledge gained from this study will help the country of Uganda in determining the best treatment for uncomplicated malaria.

**COST/PAYMENT**

After enrolment in the study, you will not be charged for clinic visits or treatment. Your child will not be paid for participation in the study. You will be reimbursed for transport costs to and from the clinic for any visit that your child requires.

**ALTERNATIVES TO PARTICIPATION**

Your child’s participation in this study is completely voluntary. If you decide you do not want to participate in the study or decide to withdraw your child from the study at any time and for any reason, this will not affect your child’s care at the outpatient department, where standard care for all medical problems is available. If your child has malaria he/she will receive the Ministry of Health’s recommendation of Chloroquine + sulfadoxine-pyrimethamine (Fansidar) [CQ+SP]. During the study, you will be informed promptly of any new information that may influence your willingness to continue participation in the study.

**CONSEQUENCES OF WITHDRAWAL**

Should you or the study doctors decide to withdraw your child from the study, your child will still be eligible for care, but payment for all treatment and medications will be your responsibility.

**USE OF THE RESULTS**

The findings from this study may be published in a medical journal. The study participants will not be identified by name. After the study is completed, you may request an explanation of the study results and ask which treatment your child received.

**PRIVACY INFORMATION**

We will keep the study information private. Under certain conditions, people responsible for making sure that the research is done properly may review your study records. This might include people involved with sponsoring or monitoring the study. All of these people are also required to keep your identity confidential. Otherwise, the information that identifies you will not be given out to people who are not working on the study.

**TREATMENT AND COMPENSATION FOR INJURY**

If you are injured or have questions about injuries as a result of being in the study, please contact the doctors in the study clinic and/or Dr. Moses Kamya (telephone 041-541188 or 041-533200) at Mulago Hospital, Kampala or Dr. Nathan Bakyaita (telephone 077-601579) at the Ugandan Ministry of Health. The services at the public health facility will be open to you in case of any such injury. However, neither the Uganda Malaria Surveillance Project nor the sponsors of this study have a program to cover your costs if your child is hurt or has other bad results.

**QUESTIONS**

This study has been explained to you by the study doctor and your questions were answered. If you have any other questions about the study, you may call Dr. Kamya at (telephone 041-541188 or 041-533200) at Mulago Hospital or Dr. Nathan Bakyaita (telephone 077-601579) at the Ugandan Ministry of Health.

**JOINING OF YOUR OWN FREE WILL**

PARTICIPATION IN RESEARCH IS VOLUNTARY. You have the right to refuse the participation of your child or to withdraw your child at any point in this study without penalty or loss of benefits to which you are otherwise entitled.

**WHAT YOUR SIGNATURE OR THUMBPRINT MEANS**

Your signature or thumbprint below means that you understand the information given to you about your child’s participation in the study and in this consent form. If you wish for your child to participate in this study, you should sign or place your thumbprint below. You will also be asked to sign another informed consent form for the use of stored biological specimens.

Name of Participant (printed)

Name of Parent/Guardian (printed)

Signature or Fingerprint * of Parent/Guardian Date/Time

Name of Investigator Administering Consent (printed) Position/Title

Signature of Investigator Administering Consent Date/Time

*If the parent or guardian is unable to read and/or write, an impartial witness should be present during the informed consent discussion. After the written informed consent form is read and explained to the participant, parent or guardian, and after they have orally consented to their child’s participation in the trial, and have either signed the consent form or provided their fingerprint, the witness should sign and personally date the consent form. By signing the consent form, the witness attests that the information in the consent form and any other written information was accurately explained to, and apparently understood by, the parent or guardian, and the parent or guardian freely gave that informed consent.

Name of Person Witnessing Consent (printed)

Signature of Person Witnessing Consent Date/Time

**Study number**: _______________________

**INFORMED CONSENT FOR FUTURE USE OF BIOLOGICAL SPECIMENS**

**Protocol Title:** Comparison of amodiaquine + artesunate and artemether + lumefantrine for treatment of uncomplicated malaria in Uganda: evaluation of efficacy, tolerability, and safety

**Site of Research:** UMSP Sentinel Sites, Uganda

**Sponsor:** Centers for Disease Control

**Principal Investigator**: Fred Wabwire-Mangen, MBChB, DTMH, MPH, PhD

**Date:** 22 September 2004

**INTRODUCTION**

Your child or the child under your care in the case of a legal guardian (hereafter referred to as “your child”) is in this study, blood samples may be taken that may be useful for future research. These samples will be stored at Makerere University Medical School and the University of California, San Francisco. Samples may also be shared with investigators at other institutions. Organisms derived from your child’s samples may also be stored in cultures for future research.

**WHAT SAMPLES WILL BE USED FOR**

Your child’s blood and the malaria parasites in it will be used to study malaria and the response of this disease to treatment. Results of these studies will not affect your child's care.

1. These samples and/or cultures will be used for future research to learn more about malaria and other diseases.
2. Your child’s samples will be used only for research and will not be sold or used for the production of commercial products.
3. Genetic research may be performed on samples. However, no genetic information obtained from this research will be placed in your child’s medical records. These samples will be identified only by codes so that they cannot be readily identified with your child.

**LEVEL OF IDENTIFICATION**

Your child’s samples will be coded so that your child’s name cannot be readily identified. Reports about research done with the samples will not be put in the medical record and will be kept confidential to the best of our ability.

In the future, researchers studying your child’s samples may need to know more about your child, such as information about age, gender, and race. If this information is already available because of your child’s participation in a study, it may be provided to the researcher. Your child’s name or anything that might identify you/them personally will not be provided. You will not be asked to provide additional consent.

**RISKS**

There are few risks to your child from future use of the samples. A potential risk might be the release of information from your child’s health or study records. Reports about research done with your child’s samples will not be put in the health record, but will be kept with the study records. The study records will be kept confidential as far as possible.

**BENEFITS**

There will be no direct benefit to your child. From studying your child’s samples we may learn more about malaria or other diseases: how to prevent them, how to treat them, how to cure them.

**RESEARCH RESULTS/MEDICAL RECORDS**

##

1. Results from future research using your child’s samples and/or cultures may be presented in publications and meetings but patient names will not be identified.
2. Reports from future research done with your child’s samples and/or cultures will not be given to you or the doctor. These reports will not be put in your child’s medical record.

**QUESTIONS**

The future use of your child’s specimens and cultures has been explained to you by the person who signed below and your questions were answered. If you have any other questions about the information here, you may call Dr. Kamya at (telephone 041-541188 or 041-533200) at Mulago Hospital or Dr. Nathan Bakyaita (telephone 077-601579) at the Ugandan Ministry of Health.

**FREEDOM TO REFUSE**

You can change your mind at any time about allowing your child’s samples to be used for future research. If you do, contact Dr. Moses Kamya (telephone 041-541188 or 041-533200) or Dr. Nathan Bakyaita (telephone 077-601579) at the Ugandan Ministry of Health. Then your child’s samples will no longer be made available for research and will be destroyed. Whether or not you allow us to use your child’s samples in future research will not have any effect on your child’s participation in this study or future participation in other studies.

**WHAT YOUR SIGNATURE OR THUMBPRINT MEANS**

Your signature or thumbprint below means that you understand the information given to you in this consent form about your child’s specimens and cultures to be used for future research. If you wish to allow your child’s specimens and cultures to be used for future research, you should sign or place your thumbprint below.

Name of Participant (printed)

Name of Parent/Guardian (printed)

Signature or Fingerprint * of Parent/Guardian Date/Time

Name of Investigator Administering Consent (printed) Position/Title

Signature of Investigator Administering Consent Date/Time

*If the parent or guardian is unable to read and/or write, an impartial witness should be present during the informed consent discussion. After the written informed consent form is read and explained to the participant, parent or guardian, and after they have orally consented to their child’s participation in the trial, and have either signed the consent form or provided their fingerprint, the witness should sign and personally date the consent form. By signing the consent form, the witness attests that the information in the consent form and any other written information was accurately explained to, and apparently understood by, the parent or guardian, and the parent or guardian freely gave that informed consent.

Name of Person Witnessing Consent (printed)

Signature of Person Witnessing Consent Date/Time

# Appendix 6a. Weight-based administration of study medications for AQ+AS group

| **Weight (kg)** | **Amodiaquine (AQ)**  **200 mg tabs (Base)** | | | **Artesunate (AS)**  **50 mg tabs** | | | **Placebo tablets** | | |
| --- | --- | --- | --- | --- | --- | --- | --- | --- | --- |
|  | **Day 0** | **Day 1** | **Day 2** | **Day 0** | **Day 1** | **Day 2** | **Day 0** | **Day 1** | **Day2** |
|  | **am** | **am** | **am** | **am** | **am** | **am** | **pm** | **pm** | **pm** |
| 10 | ½ | ½ | ¼ | ¾ | ¾ | ¾ | 1 | 1 | 1 |
| 11 | ½ | ½ | ¼ | 1 | 1 | 1 | 1 | 1 | 1 |
| 12 | ½ | ½ | ½ | 1 | 1 | 1 | 1 | 1 | 1 |
| 13 | ½ | ½ | ½ | 1 | 1 | 1 | 1 | 1 | 1 |
| 14 | ¾ | ½ | ½ | 1 ¼ | 1 ¼ | 1 ¼ | 1 | 1 | 1 |
| 15 | ¾ | ½ | ½ | 1 ¼ | 1 ¼ | 1 ¼ | 2 | 2 | 2 |
| 16 | ¾ | ¾ | ½ | 1 ¼ | 1 ¼ | 1 ¼ | 2 | 2 | 2 |
| 17 | ¾ | ¾ | ½ | 1 ½ | 1 ½ | 1 ½ | 2 | 2 | 2 |
| 18 | 1 | ¾ | ½ | 1 ½ | 1 ½ | 1 ½ | 2 | 2 | 2 |
| 19 | 1 | ¾ | ½ | 1 ½ | 1 ½ | 1 ½ | 2 | 2 | 2 |
| 20 | 1 | 1 | ½ | 1 ¾ | 1 ¾ | 1 ¾ | 2 | 2 | 2 |
| 21 | 1 | 1 | ½ | 1 ¾ | 1 ¾ | 1 ¾ | 2 | 2 | 2 |
| 22 | 1 ¼ | 1 | ½ | 1 ¾ | 1 ¾ | 1 ¾ | 2 | 2 | 2 |
| 23 | 1 ¼ | 1 | ½ | 2 | 2 | 2 | 2 | 2 | 2 |
| 24 | 1 ¼ | 1 ¼ | ½ | 2 | 2 | 2 | 2 | 2 | 2 |
| 25 | 1 ¼ | 1 ¼ | ½ | 2 | 2 | 2 | 3 | 3 | 3 |
| 26-27 | 1 ¼ | 1 | 1 | 2 ¼ | 2 ¼ | 2 ¼ | 3 | 3 | 3 |
| 28 | 1 ¼ | 1 ¼ | 1 | 2 ¼ | 2 ¼ | 2 ¼ | 3 | 3 | 3 |
| 29-31 | 1 ¼ | 1 ¼ | 1 | 2 ½ | 2 ½ | 2 ½ | 3 | 3 | 3 |
| 32 | 1 ½ | 1 ½ | 1 | 2 ½ | 2 ½ | 2 ½ | 3 | 3 | 3 |
| 33 | 1 ½ | 1 ½ | 1 | 2 ¾ | 2 ¾ | 2 ¾ | 3 | 3 | 3 |
| 34 | 1 ½ | 1 ½ | 1 ¼ | 2 ¾ | 2 ¾ | 2 ¾ | 3 | 3 | 3 |
| 35 | 1 ½ | 1 ½ | 1 ¼ | 2 ¾ | 2 ¾ | 2 ¾ | 4 | 4 | 4 |
| 36-37 | 1 ½ | 1 ½ | 1 ¼ | 3 | 3 | 3 | 4 | 4 | 4 |
| 38 | 2 | 1 ½ | 1 ¼ | 3 | 3 | 3 | 4 | 4 | 4 |
| 39 | 2 | 1 ½ | 1 ¼ | 3 ¼ | 3 ¼ | 3 ¼ | 4 | 4 | 4 |
| 40-43 | 2 | 2 | 1 | 3 ¼ | 3 ¼ | 3 ¼ | 4 | 4 | 4 |
| 44-47 | 2 | 2 | 1 ½ | 3 ½ | 3 ½ | 3 ½ | 4 | 4 | 4 |
| 48-49 | 2 | 2 | 2 | 3 ¾ | 3 ¾ | 3 ¾ | 4 | 4 | 4 |
| 50-59 | 2 ½ | 2 | 2 | 4 | 4 | 4 | 4 | 4 | 4 |
| >60 | 3 | 3 | 1 ½ | 5 | 5 | 5 | 4 | 4 | 4 |

# Appendix 6b. Weight-based administration of study medications for coartemether group

| **Weight (kg)** | **Placebo tablets** | | | **Coartemether**  **20mg /120 mg tabs** | | | | | |
| --- | --- | --- | --- | --- | --- | --- | --- | --- | --- |
|  | **Day 0** | **Day 1** | **Day 2** | **Day 0** | | **Day 1** | | **Day 2** | |
|  | am | am | am | am | pm | am | pm | am | pm |
| 10 | ½ | ½ | ¼ | 1 | 1 | 1 | 1 | 1 | 1 |
| 11 | ½ | ½ | ¼ | 1 | 1 | 1 | 1 | 1 | 1 |
| 12 | ½ | ½ | ½ | 1 | 1 | 1 | 1 | 1 | 1 |
| 13 | ½ | ½ | ½ | 1 | 1 | 1 | 1 | 1 | 1 |
| 14 | ¾ | ½ | ½ | 1 | 1 | 1 | 1 | 1 | 1 |
| 15 | ¾ | ½ | ½ | 2 | 2 | 2 | 2 | 2 | 2 |
| 16 | ¾ | ¾ | ½ | 2 | 2 | 2 | 2 | 2 | 2 |
| 17 | ¾ | ¾ | ½ | 2 | 2 | 2 | 2 | 2 | 2 |
| 18 | 1 | ¾ | ½ | 2 | 2 | 2 | 2 | 2 | 2 |
| 19 | 1 | ¾ | ½ | 2 | 2 | 2 | 2 | 2 | 2 |
| 20 | 1 | 1 | ½ | 2 | 2 | 2 | 2 | 2 | 2 |
| 21 | 1 | 1 | ½ | 2 | 2 | 2 | 2 | 2 | 2 |
| 22 | 1 ¼ | 1 | ½ | 2 | 2 | 2 | 2 | 2 | 2 |
| 23 | 1 ¼ | 1 | ½ | 2 | 2 | 2 | 2 | 2 | 2 |
| 24 | 1 ¼ | 1 ¼ | ½ | 2 | 2 | 2 | 2 | 2 | 2 |
| 25 | 1 ¼ | 1 ¼ | ½ | 3 | 3 | 3 | 3 | 3 | 3 |
| 26-27 | 1 ¼ | 1 | 1 | 3 | 3 | 3 | 3 | 3 | 3 |
| 28 | 1 ¼ | 1 ¼ | 1 | 3 | 3 | 3 | 3 | 3 | 3 |
| 29-31 | 1 ¼ | 1 ¼ | 1 | 3 | 3 | 3 | 3 | 3 | 3 |
| 32 | 1 ½ | 1 ½ | 1 | 3 | 3 | 3 | 3 | 3 | 3 |
| 33 | 1 ½ | 1 ½ | 1 | 3 | 3 | 3 | 3 | 3 | 3 |
| 34 | 1 ½ | 1 ½ | 1 ¼ | 3 | 3 | 3 | 3 | 3 | 3 |
| 35 | 1 ½ | 1 ½ | 1 ¼ | 4 | 4 | 4 | 4 | 4 | 4 |
| 36-37 | 1 ½ | 1 ½ | 1 ¼ | 4 | 4 | 4 | 4 | 4 | 4 |
| 38 | 2 | 1 ½ | 1 ¼ | 4 | 4 | 4 | 4 | 4 | 4 |
| 39 | 2 | 1 ½ | 1 ¼ | 4 | 4 | 4 | 4 | 4 | 4 |
| 40-43 | 2 | 2 | 1 | 4 | 4 | 4 | 4 | 4 | 4 |
| 44-47 | 2 | 2 | 1 ½ | 4 | 4 | 4 | 4 | 4 | 4 |
| 48-49 | 2 | 2 | 2 | 4 | 4 | 4 | 4 | 4 | 4 |
| 50-59 | 2 ½ | 2 | 2 | 4 | 4 | 4 | 4 | 4 | 4 |
| >60 | 3 | 3 | 1 ½ | 4 | 4 | 4 | 4 | 4 | 4 |

# APPENDIX 7. CLASSIFICATION OF RESPONSE TO TREATMENT

**Early Treatment Failure (ETF)**

| **ETF**   - Development of danger signs or severe malaria on Day 1, Day 2 or Day 3 in the presence of parasitemia - Parasitaemia on day 2 higher than Day 0 count irrespective of axillary temperature. - Parasitaemia on Day 3 with axillary temperature > 37.5 ^0^C - Parasitaemia on Day 3 > 25% of count on Day 0. - Severe AE requiring change in treatment Days 0 - 2 |
| --- |

**Late Treatment Failure (LTF)**

| **Late Clinical Failure (LCF):**   - Development of danger signs or severe malaria after Day 3 in the presence of parasitemia, without previously meeting any of the criteria of early treatment failure - Presence of parasitaemia and axillary temperature > 37.5°C, (or history of fever in past 24 hours), on any day from Day 4 to Day 28, without previously meeting any of the criteria of early treatment failure   **Late Parasitological Failure (LPF):**   - Presence of parasitemia on Day 28 and axillary temperature < 37.5°C, without previously meeting any of the criteria of early treatment failure or late clinical failure |
| --- |

Adequate Clinical and Parasitological Response (ACPR)

| **ACPR**   - Absence of parasitemia on Day 28 irrespective of temperature without previously meeting any of the criteria of early treatment failure or late clinical failure or late parasitological failure. |
| --- |

# Appendix 8. Criteria for Severe Malaria/Danger Signs

**Severe Malaria**

- Unarousable coma *(if after convulsion, > 30 min)*
- Repeated convulsions *(> 2 within 24 h)*
  - Severe anaemia *(Hb < 5.0 g/dL)*
  - Respiratory distress *(laboured breathing at rest)*
  - Jaundice *(yellow coloring of eyes)*

**Danger Signs**

- Recent convulsions *(1-2 within 24 h)*
- Altered consciousness  *(confusion, delerium, psychosis)*
- Lethargy
  - Unable to drink or breast feed
  - Vomiting everything
  - Unable to stand/sit due to weakness

# Appendix 9. Guidelines for Grading Patient Symptoms, signs and laboratory findings.

## Table A. Guidelines for grading patient symptoms.

|  | **Grade 1**  **MILD** | **Grade 2**  **MODERATE** | **Grade 3**  **SEVERE** | **Grade 4**  **LIFE THREATENING** |
| --- | --- | --- | --- | --- |
| **Subjective fever in the past 24 h** | N/A | Present (Yes) | N/A | N/A |
| **Weakness** | Mild decrease in activity; For children – weak, but still playing | Moderate decrease in activity; For children – weak, and playing limited | Not participating in usual activities; For children – not playing | Prostration |
| **Muscle and/or joint aches*** | Mild and/or localized complaints | Diffuse complaints | Objective weakness; function limited | N/A |
| **Headache*** | Mild, no treatment required | Transient, moderate; treatment required | Severe, constant; requires narcotic therapy | Intractable; requires repeated narcotic therapy |
| **Anorexia** | Decreased appetite, but still taking solid food | Decreased appetite, avoiding solid food but taking liquids | Appetite very decreased; Refusing to breast feed, no solids or liquids taken (< 2 years < 12 hr; > 2 years < 24 hr) | Appetite very decreased; Refusing to breast feed, no solids or liquids taken (< 2 years > 12 hr; > 2 years > 24 hr) |
| **Nausea*** | Mild, transient feeling of impending vomiting; maintains reasonable intake | Moderate and/or constant feeling of impending vomiting; intake decreased | Severe, constant feeling of impending emesis; intake decreased significantly | N/A |
| **Vomiting** | 1 episode per day | 2-3 episodes per day | Orthostatic hypotension or IV fluids required | Hypotensive shock or 42nrolment42ation required for IV fluid therapy |
| **Abdominal pain*** | Mild (1-3 on a scale of 1 to 10) | Moderate (4-6 on a scale of 1 to 10) | Moderate to severe (> 7 on a scale of 1 to 10) | Severe – 42nrolment42at for treatment |
| **Diarrhea** | Transient 3-4 loose stools/day | 5-7 loose stools/day | Orthostatic hypotension or > 7 loose stools/day or IV fluids required | Hypotensive shock or 42nrolment42ation for IV fluid therapy required |
| **Cough** | Transient / intermittent | Persistent / constant | Uncontrolled | Cyanosis, stridor, severe shortness of breath |
| **Pruritis** | Transient pruritis | Pruritis that disturbs sleep | Severe, constant pruritis, sleep disturbed | N/A |
| **Tinnitus*** | Mild, transient ringing or roaring sound | Moderate, persistent ringing or roaring sound | Severe ringing or roaring sound with associated hearing loss | N/A |
| **Behavioural changes** | Mild difficulty concentrating; mild confusion or agitation; activities of daily living unaffected; no treatment | Moderate confusion or agitation; some limitation of activities of daily living; minimal treatment | Severe confusion or agitation; Needs assistance for activities of daily living; therapy required | Toxic psychosis; 42nrolment42ation required |
| **“Flu”**  **(viral URI)** | Mild nasal congestion, mild rhinorrhea | Moderate nasal congestion, moderate rhinorrhea | N/A | N/A |
| **Allergic reaction** | N/A | N/A | Urticaria | Severe urticaria  anaphylaxis, angioedema |
| **Convulsion** | N/A | N/A | Localized or generalized seizure | Status epilepticus |
| *** Assess only in children > 3 years of age. Answer N/A for younger children and those unable to answer.** | | | | |

Reference – Based on WHO Toxicity Grading Scale for Determining the Severity of Adverse Events

## Table B. Guidelines for Physical Examination

| Dehydration | Assess skin touch and turgor, mucous membranes, eyes, crying, fontanelle, pulse, urine output |
| --- | --- |
| **Jaundice** | Assess for yellowing of the sclera. Also evaluate the palpepral conjunctiva, lips, and skin. |
| **Chest** | Observe the rate, rhythm, depth, and effort of breathing. Check the patient’s colour for cyanosis.  The maximum acceptable respiratory rate by age: < 2 months = 60, 2-12 months = 50, 1-5 years = 40, above 5 years = 30.  Inspect the neck for the position of the trachea, for supraclavicular retractions, and for contraction of the sternomastoid or other accessory muscles during inspiration.  Auscultate the anterior and posterior chest for normal breath sounds and any adventitious sounds (crackles or rales, wheezes, and rhonchi). *Crackles are intermittent, non-musical, fine or coarse sounds that may be due to abnormalities of the lungs (pneumonia, fibrosis, early congestive heart failure) or airways (bronchitis or bronchiectasis). Wheezes are high-pitched and result from narrowed airways. Rhonchi are relatively low-pitched and suggest secretions in large airways.*  If abnormalities are identified, evaluate for transmitted voice sounds. In addition, palpate the chest to assess for tactile fremitus, and percuss the chest to assess for areas of dullness*. Normal, air-filled lungs emit predominantly vesicular breath sounds, transmit voice sounds poorly with “ee” = “ee”, and have no tactile fremitus. Airless lung, as in lobar pneumonia, emits bronchial breath sounds, transmits spoken words clearly with “ee” = “aay” (egophany), and has an increase in tactile fremitus.* |
| **Abdomen** | Inspect and ausculate the abdomen. Listen for bowel sounds in the abdomen before palpating it. Palpate the abdomen in all 4 quadrants lightly and then deeply. Assess the size of the liver and spleen. To assess for peritoneal inflammation, look for localised and rebound tenderness, and voluntary or involuntary rigidity. |
| **Skin** | Inspect the skin for colour, turgor, moisture, and lesions. If lesions are present, note their location and distribution (diffuse or localised), arrangement (linear, clustered, annular, dermatomal), type (macules, papules, vesicles) and colour. |
| **Tablet test** | For children > 9 months of age, ask the patient to pick a tablet (or equivalent object) up off a flat surface using the thumb and index finger of their dominant hand*. This tests for co-ordination of the upper extremity assessing the function of the motor system, cerebellar system, vestibular system (for coordinating eye and body movements) and the sensory system, for position sense. When testing small children, be aware that they will likely attempt to put the object into their mouth.* |

## Table C. Grading Physical Examination Findings

|  | **Grade 1**  **MILD** | **Grade 2**  **MODERATE** | **Grade 3**  **SEVERE** | **Grade 4**  **LIFE-THREATENING** |
| --- | --- | --- | --- | --- |
| **Temperature* (axillary)** | 37.5-37.9°C | 38.0-39.5°C | > 39.5°C | Sustained fever, equal or greater than 40.0°C for longer than 5 days |
| **Dehydration** | Less than 2 of the following:  Restless, irritable  Sunken eyes  Drinks eagerly, thirsty  Skin pinch goes back slowly | 2 of the following:  Restless, irritable  Sunken eyes  Drinks eagerly, thirsty  Skin pinch goes back slowly | Two of the following:  Lethargic or unconscious  Sunken eyes  Not able to drink or drinking poorly  Skin pinch goes back very poorly | Two of the following + shock:  Lethargic or unconscious  Sunken eyes  Not able to drink or drinking poorly  Skin pinch goes back very poorly |
| **Jaundice** | Slight yellowing of sclera and conjunctiva | Moderate yellowing of sclera and conjunctiva, yellowing of mucous membranes | Severe yellowing of sclera and conjunctiva, yellowing of skin | N/A |
| **Chest** | Mildly increased RR (for age, temperature), transient or localised adventitious sounds | Moderately increased RR, diffuse or persistent adventitious sounds | Rapid RR (< 2 months > 60, 2-12 months > 50, 1-5 years > 40, adults > 30)* nasal flaring, retractions | Cyanosis |
| **Abdomen** | Normal bowel sounds, mild localised tenderness, and/or liver palpable 2-4 cm below the right costal margin (RCM), and/or spleen palpable, and/or umbilical hernia present | Normal or mildly abnormal bowel sounds, moderate or diffuse tenderness; and/or mild to moderately enlarged liver (4-6 cm below the RCM) and/or spleen palpable up to half-way between umbilicus and symphysis pubis | Severely abnormal bowel sounds, severe tenderness to palpation. Evidence of peritoneal irritation and/or significant enlargement of liver (> 6 cm below the RCM) and/or spleen palpable beyond half-way between umbilicus and symphysis pubis | Absent bowel sounds. Involuntary rigidity |
| **Skin†** | Localised rash, erythema, or pruritis | Diffuse, maculopapular rash, dry desquamation | Vesiculation, moist desquamation, or ulceration | Exfoliative dermatitis, mucous membrane involvement or erythema multiforme or suspected Stevens-Johnson or necrosis requiring surgery |

|  | **Grade 1**  **MILD** | **Grade 2**  **MODERATE** | **Grade 3**  **SEVERE** | **Grade 4**  **LIFE-THREATENING** |
| --- | --- | --- | --- | --- |
| **Hearing** | *< 4 years: N/A*  > 4 years: Decreased hearing in one ear | *< 4 years: N/A*  > 4 years: Decreased hearing in both ears or severe impairment in one ear | *< 4 years: Any evidence of hearing impairment*  > 4 years: Severe impairment in both ears | N/A |
| **Tablet test** | Difficulty grasping tablet but able to pick up | Unable to pick up tablet without dropping | Unable to grasp tablet | N/A |
| **Clinical symptoms / sign *(not otherwise specified)*** | No treatment required; monitor condition | Treatment required | Requires treatment and possible hospitalisation | Requires active medical intervention, hospitalisation, or hospice care |

- Reference – The Harriet Lane Handbook, 15^th^ edition, 2000

† Reference – WHO Toxicity Grading Scale for Determining the Severity of Adverse Events

## TABLE D. Guidelines for Grading of Laboratory Abnormalities

|  | **Grade 1**  **MILD** | **Grade 2**  **MODERATE** | **Grade 3**  **SEVERE** | **Grade 4**  **LIFE-THREATENING** |
| --- | --- | --- | --- | --- |
| **Haemoglobin**  ***(****g/dL)* | 9.0 – 9.9 | 7.0 – 8.9 | 5.0 – 6.9 | < 5.0 |

Reference – The Harriet Lane Handbook, 15^th^ edition, 2000†

Reference – WHO Toxicity Grading Scale for Determining the Severity of Adverse Events

# Appendix 10. Adverse Event - Follow-up Report

| **ADVERSE EVENT FORM – FOLLOW-UP REPORT** | | |
| --- | --- | --- |
| **1. Study**  **Number: U**|___|___|___|___|___| | **2. Day 0 Date:** |___|___|/|___|___|/|___|___|  ***day month year*** | **3. Treatment**  **Number:**  |___|___|___| |

| Date of follow-up: |___|___|/|___|___|/|___|___|  *day month year* | | | Study Day: | Temp: | |
| --- | --- | --- | --- | --- | --- |
| Progress Note:  _______________________________________________________  _______________________________________________________  _______________________________________________________  _______________________________________________________  _______________________________________________________  _______________________________________________________  _______________________________________________________  _______________________________________________________  _______________________________________________________  _______________________________________________________  _______________________________________________________  _______________________________________________________  _______________________________________________________  _______________________________________________________  _______________________________________________________  _______________________________________________________  _______________________________________________________ | | | Laboratory results / Other comments: | | |
| Date of follow-up: |___|___|/|___|___|/|___|___|  *day month year* | | | Study Day: | | Temp: |
| Progress Note:  _______________________________________________________  _______________________________________________________  _______________________________________________________  _______________________________________________________  _______________________________________________________  _______________________________________________________  _______________________________________________________  _______________________________________________________  _______________________________________________________  _______________________________________________________  _______________________________________________________  _______________________________________________________  _______________________________________________________  _______________________________________________________  _______________________________________________________  _______________________________________________________  _______________________________________________________ | | | Laboratory results / Other comments: | | |
| Outcome:   Resolved   Ongoing   Died | If resolved, date of resolution:  |___|___|/|___|___|/|___|___|___|___|  *day month year* | Investigator’s signature:  ________________________________  Date:________________________________ | | | |

# Appendix 11. Serious Adverse Event Form – Initial report.

| **SERIOUS adverse even form – initial report** | | |
| --- | --- | --- |
| **1. Study**  **Number: U**|___|___|___|___|___| | **2. Day 0 Date:** |___|___|/|___|___|/|___|___|  ***day month year*** | **3. Treatment**  **Number:**  |___|___|___| |

| Event description:______________________________________________________________________________  *(symptom, sign, or laboratory abnormality)* | | | | |
| --- | --- | --- | --- | --- |
| Date of event onset:  |___|___|/|___|___|/|___|___|___|___|  day month year | | Date event reported:  |___|___|/|___|___|/|___|___|___|___|  day month year | | Indicate reason for serious AE:   Fatal   Life-threatening   Resulted in significant /  persistent disability or  incapacity   Resulted in hospitalization   Prolonged hospitalization   Required medical / surgical  intervention to prevent serious  outcome   Other:____________________ |
| Maximum event severity:   Moderate   Severe   Life-threatening | | Maximum relationship to study drugs:   None   Unlikely   Possible   Probable   Definite | |  |
| Was the event unexpected?  ____ Yes ____No | |  |  |  |
| Clinical history:  ________________________________________  ________________________________________  ________________________________________  ________________________________________  ________________________________________  ________________________________________  ________________________________________  ________________________________________  ________________________________________  ________________________________________  ________________________________________  ________________________________________  ________________________________________  ________________________________________  ________________________________________  ________________________________________  ________________________________________  ________________________________________  ________________________________________  ________________________________________  ________________________________________  ________________________________________ | | | Relevant past medical history:  ________________________________________  ________________________________________  ________________________________________  ________________________________________  ________________________________________ | |
|  |  |  | Concomitant medications:  1.__________________________________________  2.__________________________________________  3.__________________________________________  4.__________________________________________  5.__________________________________________ | |
|  |  |  | Action taken: (tick all that apply)   No change in current management   Study medication discontinued   Specific treatment given   Patient hospitalized   Laboratory tests obtained   Other:____________________   Other:____________________ | |
| Date form completed:  |___|___|/|___|___|/|___|___|___|___|  *day month year* | Investigator’s name (printed): ________________________________  Investigator’s signature: ______________________________________ | | | |

# Appendix 12. Clinical Record forms

| **UMSP clinical record form (1):** | | | | | | | | |
| --- | --- | --- | --- | --- | --- | --- | --- | --- |
| **Patient**  **Initials:** | **1. Study Number: U**|___|___|___|___|___| | | **2. Day 0 Date:** |___|___|/|___|___|/|___|___|  ***day month year*** | | | | **3. Treatment Number:** |___|___|___| | |
| **4. Age:_______years______months**  *(include months only if age < 5 years, else write “X”)* | | **5. Gender: _____M _____F** | | | **6. Weight** *(kg):* | **7. Known drug allergies: Yes ___No ___Unknown ___**  **If yes, describe___________________________________** | | |
| ***List all medications taken within the last 2 weeks*** | | | | | | | | |
| **Drug** *(if name unknown, list by letter – “Unknown Drug A”)* **(a)** | | | | **Dose (b)** | | | | **Date last dose taken (c)** |
| 8. | | | | |  Complete  Incomplete  Unknown  N/A. | | --- | | | | |  |
| 9. | | | | | |  Complete  Incomplete  Unknown  N/A. | | --- | | | --- | --- | | | | |  |
| 10. | | | | | |  Complete  Incomplete  Unknown  N/A. | | --- | | | --- | --- | | | | |  |
| 11. | | | | | |  Complete  Incomplete  Unknown  N/A. | | --- | | | --- | --- | | | | |  |

| **symptom record**  (Rank on scale of 0-4: absent = 0; mild = 1; moderate = 2; severe = 3, life-threatening = 4, N/A = unable to assess) | | | | | | | | | | |
| --- | --- | --- | --- | --- | --- | --- | --- | --- | --- | --- |
|  | **day 0 (a)** | **day 1 (b)** | **day 2 (c)** | **day 3 (d)** | **day 7 (e)** | **day 14 (f)** | **day 21 (g)** | **day 28 (h)** | **day ---- (i)** | **day ---- (j)** |
| **DATE** |  |  |  |  |  |  |  |  |  |  |
| 12. Subjective fever  in past 24h *(Y/N)* |  |  |  |  |  |  |  |  |  |  |
| 13. Weakness |  |  |  |  |  |  |  |  |  |  |
| 14. Muscle/joint aches* |  |  |  |  |  |  |  |  |  |  |
| 15. Headache* |  |  |  |  |  |  |  |  |  |  |
| 16. Anorexia |  |  |  |  |  |  |  |  |  |  |
| 17. Nausea* |  |  |  |  |  |  |  |  |  |  |
| 18. Vomiting |  |  |  |  |  |  |  |  |  |  |
| 19. Abdominal pain* |  |  |  |  |  |  |  |  |  |  |
| 20. Diarrhea |  |  |  |  |  |  |  |  |  |  |
| 21. Cough |  |  |  |  |  |  |  |  |  |  |
| 22. Pruritis |  |  |  |  |  |  |  |  |  |  |
| 23. Tinnutus* |  |  |  |  |  |  |  |  |  |  |
| 24. Behavioural changes |  |  |  |  |  |  |  |  |  |  |
| 25. “Flu” |  |  |  |  |  |  |  |  |  |  |
| 26. Other______________ |  |  |  |  |  |  |  |  |  |  |
| 27. Other______________ |  |  |  |  |  |  |  |  |  |  |
| 28. Adverse event  reported† *(Y/N)* |  |  |  |  |  |  |  |  |  |  |
| Initials |  |  |  |  |  |  |  |  |  |  |

****Only assess in children > 3 years of age. For children < 3 and those unable to answer, enter N/A.***

***† Adverse event reported if symptom is new or worsening and grade is > 2. Notify Kampala core facility immediately of all serious adverse events.***

| **UMSP clinical record form (2):** | | | |
| --- | --- | --- | --- |
| **Patient**  **Initials:** | **1. Study Number: U**|___|___|___|___|___| | **2. Day 0 Date:** |___|___|/|___|___|/|___|___|  ***day month year*** | **3. Treatment Number:** |___|___|___| |

| **physical exam record**  (Rank on scale of 0-4: normal = 0; mild abnormality = 1; moderate = 2; severe = 3, life-threatening = 4, N/A = unable to assess) | | | | | | | | | | |
| --- | --- | --- | --- | --- | --- | --- | --- | --- | --- | --- |
|  | **day 0 (a)** | **day 1 (b)** | **day 2 (c)** | **day 3 (d)** | **day 7 (e)** | **day 14 (f)** | **day 21 (g)** | **day 28 (h)** | **day ---- (i)** | **day ---(j)** |
| **DATE** |  |  |  |  |  |  |  |  |  |  |
| 29. Temperature *(ºC)* |  |  |  |  |  |  |  |  |  |  |
| 30. Dehydration |  |  |  |  |  |  |  |  |  |  |
| 31. Jaundice |  |  |  |  |  |  |  |  |  |  |
| 32. Chest |  |  |  |  |  |  |  |  |  |  |
| 33. Abdomen |  |  |  |  |  |  |  |  |  |  |
| 34. Skin |  |  |  |  |  |  |  |  |  |  |
| 35. Hearing |  |  |  |  |  |  |  |  |  |  |
| 36. Tablet test* |  |  |  |  |  |  |  |  |  |  |
| 37. Other__________ |  |  |  |  |  |  |  |  |  |  |
| 38. Other__________ |  |  |  |  |  |  |  |  |  |  |
| 39. Adverse event  reported† *(Y/N)* |  |  |  |  |  |  |  |  |  |  |
| ABNORMAL EXAM RECORD | | | | | | | | | | |
| If abnormality noted on physical exam, describe all physical findings for the abnormal exam |  |  |  |  |  |  |  |  |  |  |
| **Initials** |  |  |  |  |  |  |  |  |  |  |

**** Follow age-based guidelines: Tablet test – > 9 mo; Heel-toe – > 2 years; Romberg – > 4 years. Answer N/A for younger children and uncooperative patients.***

***† Adverse event reported if exam sign is new or worsening and grade is > 2. Notify Kampala core facility immediately of all serious adverse events.***

| **UMSP clinical record form (3):** | | | |
| --- | --- | --- | --- |
| **Patient**  **Initials:** | **1. Study Number: U**|___|___|___|___|___| | **2. Day 0 Date:** |___|___|/|___|___|/|___|___|  ***day month year*** | **3. Treatment Number:** |___|___|___| |

| LABORATORY RECORD | | | | | | | | | | |
| --- | --- | --- | --- | --- | --- | --- | --- | --- | --- | --- |
|  | **day 0 (a)** | **day 1 (b)** | **day 2 (c)** | **day 3 (d)** | **day 7 (e)** | **day 14 (f)** | **day 21(g)** | **day 28 (h)** | **day ---- (i)** | **day ---- (j)** |
| **DATE** |  |  |  |  |  |  |  |  |  |  |
| 40. Parasite density  *(asexual parasites/ul)* |  |  |  |  |  |  |  |  |  |  |
| 41. Species |  |  |  |  |  |  |  |  |  |  |
| 42. Gametocytes *(Y/N)* |  |  |  |  |  |  |  |  |  |  |
| 43. Haemoglobin*† *(g/dL)*  [grade] | [ ] |  |  |  |  |  |  | [ ] |  |  |
| Initials |  |  |  |  |  |  |  |  |  |  |

****(Grade on scale of 0-4: normal = 0; mild abnormality = 1; moderate = 2; severe = 3, life-threatening = 4)***

***† Any haemoglobin <*** 5g/dl measured after Day 0 is a serious AE. Notify Kampala core facility immediately of all serious adverse events.

| **RECORD OF ADDITIONAL MEDICATION GIVEN DURING STUDY** | | | | |
| --- | --- | --- | --- | --- |
| **Medication (a)** | **Indication (b)** | **Dose (c)** | **Duration (d)** | **Date started (e)** |
| **60.** |  |  |  |  |
| **61.** |  |  |  |  |
| **62.** |  |  |  |  |
| **63.** |  |  |  |  |
| **64.** |  |  |  |  |
| **65.** |  |  |  |  |
| 66. |  |  |  |  |
| **67.** |  |  |  |  |
| **68.** |  |  |  |  |
| **69.** |  |  |  |  |
| **70.** |  |  |  |  |

| **UMSP clinical record form (4):** | | | |
| --- | --- | --- | --- |
| **Patient**  **Initials:** | **1. Study Number: U**|___|___|___|___|___| | **2. Day 0 Date:** |___|___|/|___|___|/|___|___|  ***day month year*** | **3. Treatment Number:** |___|___|___| |

| complete efficacy outcome | |
| --- | --- |
|  ETF     LCF   LPF   ACPR     N/A (Tick appropriate Incomplete Efficacy Outcome **)** | If failed, **STUDY DAY** of clinical failure (0-28) __________  **Reason for Clinical Failure:**   Severe malaria/danger signs with parasitemia Days 0-3 🡪 Specify criteria _________   Severe AE requiring change in treatment Days 0-2 🡪 Specify criteria _____________   Day 2 parasite count > Day 0 count   Parasitemia on Day 3 with temperature > 37. 5   Day 3 parasite count > 25% Day 0 count   Severe malaria with parasitemia Days 4-28 🡪 Specify criteria __________________   Parasitemia on Days 4-28 with temperature > 37. 5 OR History of fever in past 24 hours. |

**Outcome Classification**

**ETF🡪** Assessed Days 0-3

**LCF🡪** Assessed Days 4-28 and previously not an ETF.

**LPF🡪** Assessed Day28

and previously not an ETF

or LCF.

**ACPR🡪** Assessed Day28 and previously not an ETF, or LCF.

**For exact definitions refer to:**

- Study Poster in Clinic
- Field Manual Appendix 3
- SOP # DATA3-003 CRF

| INCOMPLETE EFFICACY OUTCOME |
| --- |
|  **Excluded** - If yes, reason for exclusion: (Must tick one reason below)   Other antimalarial use: if yes, describe__________________________   Withdrew informed consent   Concomitant febrile illness: if yes, diagnosis_____________________   Lost - If yes, last Study day examined: _____________   Error made during follow-up that prevented outcome classification: ______________________________ |

**Appendix 16. UMSP Adverse Event Record Form**

| **adverse event record form** | | | |
| --- | --- | --- | --- |
| **Patient**  **Initials:** | **1. Study Number: U**|___|___|___||___||___| | **2. Day 0 Date:** |___|___|/|___|___|/|___|___|  ***day month year*** | **3. Treatment Number:** |___|___|___| |

|  | ***Complete on day first reported*** | | | ***Complete on day first reported and update as needed*** | | | ***Complete on final day*** | | |
| --- | --- | --- | --- | --- | --- | --- | --- | --- | --- |
| **Event description (a)** | **Date of event onset (b)** | **Date event reported**  **(c)** | **Initials of person reporting** | **Maximum severity* (d)** | **Maximum relationship† (e)** | **Serious? ‡ *(Y/N)***  **(f)** | **Episodic?**  ***(Y/N)***  **(g)** | **Outcome †† (h)** | **Date event resolved‡‡ (i)** |
| **80.** |  |  |  |  |  |  |  |  |  |
| **81.** |  |  |  |  |  |  |  |  |  |
| **82.** |  |  |  |  |  |  |  |  |  |
| **83.** |  |  |  |  |  |  |  |  |  |
| **84.** |  |  |  |  |  |  |  |  |  |
| **85.** |  |  |  |  |  |  |  |  |  |
| **86.** |  |  |  |  |  |  |  |  |  |
| **87.** |  |  |  |  |  |  |  |  |  |
| **88.** |  |  |  |  |  |  |  |  |  |
| **89.** |  |  |  |  |  |  |  |  |  |
| **90.** |  |  |  |  |  |  |  |  |  |
| **91.** |  |  |  |  |  |  |  |  |  |
| **92.** |  |  |  |  |  |  |  |  |  |
| **93.** |  |  |  |  |  |  |  |  |  |
| **94.** |  |  |  |  |  |  |  |  |  |

*** d) Severity:** *Rank on scale of 1-4: mild = 1; moderate = 2; severe = 3, life-threatening = 4*

**† e) Relationship:** *Rank on scale of 0-4: none = 0; unlikely = 1; possible = 2; probable = 3; definite = 4*

**‡ f) Serious:** Criteria for serious AE: fatal, life-threatening, results in or prolongs hospitalization, results in significant or persistent disability or capacity requires medical / surgical intervention to prevent serious outcome. **If serious, report to Kampala core facility staff immediately. They will assist with patient management, assist with completion of serious AE forms, and report the AE if necessary.**

**††h) Outcome:** *Rank on scale of 1-5: resolved without sequelae = 1; resolved with sequelae = 2; AE still present at study end/discontinuation, but improving = 3; subject died = 4; unknown = 5*

**‡‡ i) Date event resolved:** *Complete on Day 28 – If AE still ongoing at end of follow-up, indicate in question (h).*
